# Diversification and Distribution of Ruminant *Chlamydia abortus* Clones Assessed by MLST and MLVA

**DOI:** 10.1371/journal.pone.0126433

**Published:** 2015-05-22

**Authors:** Victoria I. Siarkou, Fabien Vorimore, Nadia Vicari, Simone Magnino, Annie Rodolakis, Yvonne Pannekoek, Konrad Sachse, David Longbottom, Karine Laroucau

**Affiliations:** 1 Laboratory of Microbiology and Infectious Diseases, School of Veterinary Medicine, Faculty of Health Sciences, Aristotle University of Thessaloniki, Thessaloniki, Greece; 2 Anses, Animal Health Laboratory, Bacterial Zoonoses Unit, University Paris-Est, Maisons-Alfort, France; 3 Istituto Zooprofilattico Sperimentale della Lombardia e dell’Emilia Romagna “Bruno Ubertini”, Sezione Diagnostica di Pavia, Pavia, Italy; 4 INRA, Infectiologie Animale et Santé Publique, Nouzilly, France; 5 Academic Medical Center, Department of Medical Microbiology, University of Amsterdam, Amsterdam, The Netherlands; 6 Institute of Molecular Pathogenesis, Friedrich-Loeffler-Institut (Federal Research Institute for Animal Health), Jena, Germany; 7 Moredun Research Institute, Edinburgh, Midlothian, United Kingdom

## Abstract

*Chlamydia abortus*, an obligate intracellular bacterium, is the most common infectious cause of abortion in small ruminants worldwide and has zoonotic potential. We applied multilocus sequence typing (MLST) together with multiple-locus variable-number tandem repeat analysis (MLVA) to genotype 94 ruminant *C*. *abortus* strains, field isolates and samples collected from 1950 to 2011 in diverse geographic locations, with the aim of delineating *C*. *abortus* lineages and clones. MLST revealed the previously identified sequence types (STs) ST19, ST25, ST29 and ST30, plus ST86, a recently-assigned type on the *Chlamydiales* MLST website and ST87, a novel type harbouring the *hemN*_21 allele, whereas MLVA recognized seven types (MT1 to MT7). Minimum-spanning-tree analysis suggested that all STs but one (ST30) belonged to a single clonal complex, possibly reflecting the short evolutionary timescale over which the predicted ancestor (ST19) has diversified into three single-locus variants (ST86, ST87 and ST29) and further, through ST86 diversification, into one double-locus variant (ST25). ST descendants have probably arisen through a point mutation evolution mode. Interestingly, MLVA showed that in the ST19 population there was a greater genetic diversity than in other STs, most of which exhibited the same MT over time and geographical distribution. However, the evolutionary pathways of *C*. *abortus* STs seem to be diverse across geographic distances with individual STs restricted to particular geographic locations. The ST30 singleton clone displaying geographic specificity and represented by the Greek strains LLG and POS was effectively distinguished from the clonal complex lineage, supporting the notion that possibly two separate host adaptations and hence independent bottlenecks of *C*. *abortus* have occurred through time. The combination of MLST and MLVA assays provides an additional level of *C*. *abortus* discrimination and may prove useful for the investigation and surveillance of emergent *C*. *abortus* clonal populations.

## Introduction

*Chlamydia abortus* is an obligate intracellular bacterium that can colonize the placenta of several animal species causing abortion or stillbirth [1–3]. This organism also represents a threat to human health because it can cause spontaneous abortion and possible life-threatening disease in pregnant women exposed to infected animals [3]. *C*. *abortus* is endemic among small ruminants and is the most common cause of infectious abortion in sheep and goats in many countries worldwide [1].

*C*. *abortus* is classified as a member of the family *Chlamydiaceae* that currently comprises the single genus *Chlamydia*, which contains 11 species (*C*. *abortus*, *C*. *avium*, *C*. *caviae*, *C*. *felis*, *C*. *gallinacea*, *C*. *muridarum*, *C*. *pecorum*, *C*. *pneumoniae*, *C*. *psittaci*, *C*. *suis* and *C*. *trachomatis*) and a novel candidate species named *C*. *ibidis* [4–6]. Studies using different phenotypic and molecular approaches have suggested that the genetic heterogeneity of *C*. *abortus* is low. Methods based on the cross reactivity of monoclonal antibodies, restriction patterns of the *omp*A gene and the phylogenetic analysis of rRNA genes, resulted in little or no evidence of genetic diversity with regard to the host, associated disease or geographical origin of strains [7–9]. However, a more sophisticated molecular typing tool, namely amplified fragment length polymorphism analysis, enabled better discrimination of French strains from those of other origin, even when rRNA and *omp*A sequences were highly conserved [10]. Further to this, two *C*. *abortus* strains, named LLG and POS, isolated in Greece from an aborted goat and sheep, respectively [11,12], were found to be considerably different from other *C*. *abortus* strains circulating in the same area. These strains were characterized as variants on the basis of unique inclusion morphology, differences in polypeptide profiles and antibody cross-reactivity, diversity of rRNA, *omp*A and *pmp* sequences, and different behavior and ability to colonize the placenta and fetus compared to other wild-type strains [11–17]. Comparison of the genome sequence of the LLG strain with the wild-type *C*. *abortus* reference strain S26/3 revealed notable differences in the pseudogene content [18,19]. rRNA secondary structure phylogeny revealed that the two Greek variant strains could represent one distinct lineage evolving independently from other *C*. *abortus* strains, to such an extent that "subspecies" status has been suggested for them [20].

Interestingly, a recent study using multiple-locus variable-number tandem repeat (VNTR) analysis (MLVA), as well as a different approach using a multilocus sequence typing (MLST) system, has allowed the differentiation of *C*. *abortus* strains into distinct genotypes [21,22]. The MLVA typing method, based on the analysis of five VNTR loci, enabled the clustering of 145 *C*. *abortus* strains into six genotypes [21]. In contrast, MLST analysis targeting seven housekeeping genes [23], recognized four sequence types (STs) among the 16 *C*. *abortus* strains examined [22]. Having considered that MLST was evaluated on too few *C*. *abortus* strains, this study aimed to determine the suitability of MLST for genotyping *C*. *abortus* in comparison to MLVA. To achieve this, a well-referenced collection of *C*. *abortus* strains of known MLVA genotypes, along with two other collections of field isolates and samples were genotyped. In addition, we aimed to explore and delineate the *C*. *abortus* clonal lineages to obtain new insights into how clones or lineages of particular epidemiological relevance emerge and diversify.

## Materials and Methods

### *C*. *abortus* strains, field isolates and samples

In this study a total of 94 *C*. *abortus* genomic DNAs were analyzed. These comprised: (i) a collection of 33 strains (panel A) that were representative of all *C*. *abortus* genotypes, as determined by MLVA [21]; (ii) a collection of 21 isolates (panel B) randomly selected from *C*. *abortus* field isolates belonging to the predominant MLVA genotype MT2 [21]; and (iii) a collection of 40 field pathological samples (panel C) obtained from cases of abortion occurring in sheep, goats and cattle.

The *C*. *abortus* strains and isolates used in this study originated from nine countries and were collected between 1950 and 2011. Genomic DNAs were extracted (QIAamp DNA mini Kit; Qiagen) from the first or second culture passage of the original strains and isolates. Additional field samples originated from different regional veterinary diagnostic laboratories or veterinary services in France, Greece and Italy, from abortion cases between 2005 and 2011. The origin and source of the *C*. *abortus* strains, isolates and samples investigated are presented in Tables 1 and 2.

**Table 1:** MLST profile and epidemiological characteristics of 33 representative strains (panel A) of all *C*. *abortus* genotypes determined by MLVA and characterized as MTs (MT1 to MT7).

MLVA Type	Type-strain for MLVA	Host	Clinical origin	Country	MLST alleles							MLST Type
					*gatA*	*oppA*	*hflX*	*gidA*	*enoA*	*hemN*	*fumC*	
**MT1**	C9/98	sheep	abortion	De	**5**	8	6	8	8	**4**	**5**	**ST19**
**MT2**	Kra	goat	abortion	De	**5**	8	6	8	8	**4**	**18**	**ST29**
	Z178/02*	sheep	abortion	De	**18**	8	6	8	8	**14**	**5**	**ST25**
	AB1	sheep	abortion	Fr	**18**	8	6	8	8	**14**	**5**	**ST25**
	AB4	sheep	abortion	Fr	**18**	8	6	8	8	**14**	**5**	**ST25**
	AB7	sheep	abortion	Fr	**18**	8	6	8	8	**14**	**5**	**ST25**
	AB8	sheep	abortion	Fr	**18**	8	6	8	8	**14**	**5**	**ST25**
	AB15*	sheep	abortion	Fr	**18**	8	6	8	8	**14**	**5**	**ST25**
	AB16	sheep	abortion	Fr	**18**	8	6	8	8	**14**	**5**	**ST25**
	VB1	sheep	epididymitis	Fr	**18**	8	6	8	8	**14**	**5**	**ST25**
	OC1	sheep	conjunctivitis	Fr	**18**	8	6	8	8	**14**	**5**	**ST25**
	1B	-	*AB7-mutant*	Fr	**18**	8	6	8	8	**14**	**5**	**ST25**
	1H	-	*AB7-mutant*	Fr	**18**	8	6	8	8	**14**	**5**	**ST25**
	AB13	sheep	abortion	Fr	**5**	8	6	8	8	**14**	**5**	**ST86**
	AV1	cattle	abortion	Fr	**5**	8	6	8	8	**14**	**5**	**ST86**
	AC1	goat	abortion	Fr	**5**	8	6	8	8	**14**	**5**	**ST86**
	60172	goat	abortion	It	**5**	8	6	8	8	**14**	**5**	**ST86**
	38552	sheep	abortion	It	**5**	8	6	8	8	**14**	**5**	**ST86**
	Krauss-15	goat	abortion	Tu	**5**	8	6	8	8	**4**	**18**	**ST29**
	FAG	goat	abortion	Gr	**5**	8	6	8	8	**4**	**5**	**ST19**
	VPG	goat	abortion	Gr	**5**	8	6	8	8	**4**	**5**	**ST19**
**MT3**	71	sheep	intestinal	Gr	**5**	8	6	8	8	**4**	**5**	**ST19**
	B577	sheep	abortion	USA	**5**	8	6	8	8	**4**	**5**	**ST19**
	Mo907	sheep	intestinal	USA	**5**	8	6	8	8	**4**	**5**	**ST19**
	Z1215/86	cattle	abortion	De	**5**	8	6	8	8	**4**	**5**	**ST19**
**MT4**	FAS	sheep	abortion	Gr	**5**	8	6	8	8	**4**	**5**	**ST19**
	4PV	goat	abortion	It	**5**	8	6	8	8	**4**	**5**	**ST19**
	BAF	cattle	abortion	UK	**5**	8	6	8	8	**4**	**5**	**ST19**
**MT5**	A22	sheep	abortion	UK	**5**	8	6	8	8	**4**	**5**	**ST19**
	S26/3	sheep	abortion	UK	**5**	8	6	8	8	**4**	**5**	**ST19**
**MT6**	LLG	goat	abortion	Gr	**13**	18	6	19	16	**4**	**17**	**ST30**
	POS	sheep	abortion	Gr	**13**	18	6	19	16	**4**	**17**	**ST30**
**MT7**	CY71	sheep	abortion	Cy	**5**	8	6	8	8	**4**	**18**	**ST29**

* The Z178/02 and AB15 strains exhibited the 1B-vaccine-type profile; Z178/02 was recovered from a diseased animal in a vaccinated sheep flock while the AB15 strain from an unvaccinated ewe which had extensive contact with a vaccinated herd at the INRA facility in Nouzilly, in 1986, during the original vaccine trials.

The MLVA and MLST types (MTs and STs, respectively) as well as the most diverse MLST loci are indicated in bold. Country abbreviations: De, Germany; Fr, France; Gr, Greece; It, Italy; Tu, Tunisia; UK, United Kingdom; USA, United States of America; Cy, Cyprus.

**Table 2:** MLST profile based on *gatA*, hemN and fumC loci of *C*. *abortus* field isolates (n = 21; panel B) and field samples (n = 40; panel C) with the vast majority of them belonging to MT2^a^.

*C*. *abortus* field isolates	*C*. *abortus* field samples	Host	Country	MLST alleles			MLST type^*b*^
				*gatA*	*hemN*	*fumC*	
AB2, AB11	11–1100_L029, 11–1100_L030, 11–1775_Q069, 11–1775_Q070	sheep	Fr	5	4	5	**ST19**
HAS, KAS	09–772_13_74/6S, 09–772_18_81/6S, 09–772_24_88/6S, 09–772_26_41/6S	sheep	Gr	5	4	5	**ST19**
LGG, TRG	‒	goat	Gr	5	4	5	**ST19**
‒	09–772_PV4_11371/09	goat	It	5	4	5	**ST19**
11–232_Ec797, 11–232_Ec838	11–1100_L038, 11–1100_L043*, 11–1100_L045, 11–1775_Q072*, 11–1775_Q073*	sheep	Fr	18	14	5	**ST25**
‒	11–0178_E053, 11–0178_E099	cattle	Fr	18	14	5	**ST25**
SB1	‒	springbok	Fr	18	14	5	**ST25**
‒	09–772_15_15/6S^*a*^ *	sheep	Gr	18	14	5	**ST25**
‒	09–772_PV10_327435/07 ^*a*^ *	goat	It	18	14	5	**ST25**
C21/98, A-57, A-55	‒	goat	Na	5	4	18	**ST29**
15, 363, 469, 532	‒	goat	Tu	5	4	18	**ST29**
‒	09–772_3_3/5S^*a*^	sheep	Gr	5	4	18	**ST29**
‒	09–772_66_30/9G^*a*^	goat	Gr	13	4	17	**ST30**
AB22, 10–2431_Moulin56	11–1779_G003, 11–1779_G008, 11–1100_L015, 11–1100_L044, 11–1100_L047, 11–1100_L060, 11–1429_N084, 11–1697_Q034, 11–1775_Q062, 11–1775_Q064, 11–1775_Q066, 11–1775_Q067, 11–1775_Q068, 11–1775_Q071	sheep	Fr	5	14	5	**ST86**
iC1	‒	goat	Fr	5	14	5	**ST86**
‒	11–0178_E065	cattle	Fr	5	14	5	**ST86**
1107/1, 36550	09–772_PV2_7172/09, 09–772_PV3_10283/09, 09–772_PV11_2473/08, 09–772_PV9_57768/06	goat	It	5	14	5	**ST86**
‒	09–772_9_69/6S	sheep	Gr	5	21	5	**ST87**

^*a*^ All *C*. *abortus* isolates and samples typed by MLVA exhibited the genotype MT2 with exception of the 09–772_66_30/9G sample belonging to MT6; the MLVA type of the 09–772_PV10_327435/07 sample was not typeable due to the absence of ChlaAb_914 locus amplification, while the MLVA type of 09–772_15_15/6S and 09–772_3_3/5S samples was not determined due to their inadequate quantity. *C*. *abortus* field samples marked with asterisks (*) displayed the 1B-vaccine-type profile.

^*b*^ The complete MLST profile was also established for the novel STs. The MLST types (STs) are indicated in bold.

Country abbreviations: Fr, France; Gr, Greece; It, Italy; Tu, Tunisia; Na, Namibia

### Detection of *C*. *abortus* DNA and the 1B-vaccine-type profile

All DNA samples were confirmed to be *C*. *abortus* with a species-specific real-time PCR assay targeting the *omp*A gene [24] prior to genotypic characterization. Furthermore, the previously described PCR-RFLP and/or the high-resolution melt PCR (HRM-PCR) assays [25,26] were used to discriminate vaccine-strain-type from wild-type field isolates and samples.

### MLVA genotyping

MLVA genotyping was performed by targeting five tandem repeat loci, as previously described [21]. Repeats were amplified using primer sets ChlaAb_457, ChlaAb_581, ChlaAb_620, ChlaAb_914 and ChlaAb_300, and allele numbers were assigned based on fragment sizes. Numerical values were assigned for distinct MLVA types, characterized as MTs (Table 1).

### MLST genotyping

MLST genotyping targeted seven housekeeping genes, namely *gatA*, *oppA*, *hflX*, *gidA*, *enoA*, *hemN* and *fumC*, as previously described [23]. Target genes were amplified and sequenced using primers and conditions described on the *Chlamydiales* MLST website (http://pubmlst.org/chlamydiales/http://mlst.ucc.ie/). Sequencing of both DNA strands was performed by Eurofins (Germany). Numbers for alleles and sequence types (STs) were assigned in accordance with the *Chlamydiales* MLST Database.

### Assignment to clonal complex

MLST and MLVA results were entered into BioNumerics software v7.1 (Applied Maths) for minimum-spanning-tree analysis. Priority rules within the BioNumerics software were set to assign the primary founder (clonal ancestor) as the ST that initially would diversify to produce variants that differ at only one of the seven loci, as was previously described for the eBURST algorithm for inferring patterns of evolutionary descent from MLST data [27]. The clonal complex was defined as a cluster of STs, in which all STs were linked as single-locus variants to at least one other ST. STs not sharing alleles with any other ST in the dataset at six of the seven loci were assigned as singletons [27].

### Phylogenetic analysis

Maximum likelihood trees for each chlamydial MLST locus were reconstructed to determine the extent by which the phylogenetic signal varied between gene loci, testing for possible recombination [28,29]. For each unique *C*. *abortus* ST, the sequences of all seven loci were concatenated to produce an in-frame sequence of 3,098 bp, and a maximum likelihood tree was constructed. The HKY85 model of nucleotide substitution, assuming a discrete gamma distribution with eight categories, was used for tree reconstruction using MEGA5 software [30]. *dN*/*dS* ratios were computed by the method of Nei and Gojobori [31] as implemented in MEGA5 [30]. An unweighted pair group method with averages (UPGMA) dendrogram derived from three concatenated sequences of the most diverse loci was constructed to cluster the MLST and MLVA profiles in combination with their epidemiological data.

## Results and Discussion

### Assessment of *C*. *abortus* genotypes by MLVA and MLST

Initially, the MLST scheme was applied to the panel A strains to assess the genetic relatedness of strains with particular MLVA profiles. Specifically, 32 representative strains belonging to the six MLVA genotypes (MT1 to MT6) [21] were analyzed, including the mutant vaccine-1B and 1H strains (Table 1). Another vaccine strain (CY71), which was used until 2006 in the preparation of an inactivated, whole-organism adjuvanted vaccine in Cyprus, was also included as a new MLVA genotype, designated MT7 (Table 1). The CY71 strain presented with the profile 0.5-1-2-1-3 by the previously detailed VNTRs series, harbouring a new size for the ChlaAb_457 marker (S1 Fig).

MLST showed that this collection of 33 strains comprised five distinct STs, namely the previously identified ST19, ST25, ST29 and ST30 [22], plus a new allelic combination, recently designated ST86 on the *Chlamydiales* MLST website. This new allelic profile (ST86) was detected in five strains belonging to MT2 (Table 1). Interestingly, strains belonging to MT2 harboured a variety of STs (ST19, ST25, ST29 and ST86). In contrast, strains that were grouped by MLST as ST19 could be further differentiated by MLVA into MT1, MT2, MT3, MT4 and MT5. The same was also observed for ST29 that was delineated into two MTs, MT2 and MT7. The variant *C*. *abortus* strains LLG and POS [12,13,15,17,20], with distinct profile MT6 [21], also showed a distinct MLST profile (ST30). This is consistent with the previous study where, 16 *C*. *abortus* strains, including LLG and POS, were typed by MLST [22]. However, a discrepancy was observed for the strain AB7 and its mutant vaccine-1B strain in comparison with the previously reported results [22] as, in our study, these two strains were indistinguishable. This is thought to result from the use of an incorrect strain, originally thought to be "AB7", in that study. Among panel A strains, the most diverse loci were found to be *gatA*, *hemN* and *fumC*, while *oppA*, *gidA* and *enoA* exclusively differentiated variant strains LLG and POS (Table 1). Since *gatA*, *hemN* and *fumC* were responsible for a substantial part of the overall resolution obtained with the MLST system, resulting in a clustering consistent with that obtained when all loci were used (data not shown), their allelic profiles were analyzed for all field isolates and samples (Table 2). All isolates and samples genotyped by MLVA belonged to MT2, with the exception of one sample belonging to MT6. Another sample could not be genotyped due to the absence of ChlaAb_914 locus amplification. Incomplete MLVA profiles, possibly resulting from the lack of a VNTR region or sequence polymorphisms and modifications that hinder annealing of primers, have been observed in the past for other bacteria [32,33]. MLST analysis resulted in the detection of a new allelic profile, restricted to only one sample (09–772_9_69/6S in Table 2) harbouring a novel *hemN* allele, which was deposited and denoted *hemN*_21 on the *Chlamydiales* MLST website. The complete MLST profile was established for this novel ST (ST87; *gatA*_5, *oppA*_8, *hflX*_6, *gidA*_8, *enoA*_8, *hemN*_21, *fumC*_5). Altogether, six STs were identified, namely ST19, ST25, ST29, ST30, ST86 and ST87. As expected, the ST30 profile was detected and confirmed in one field sample (09–772_66_30/9G in Table 2) presenting profile MT6.

**Fig 1:**
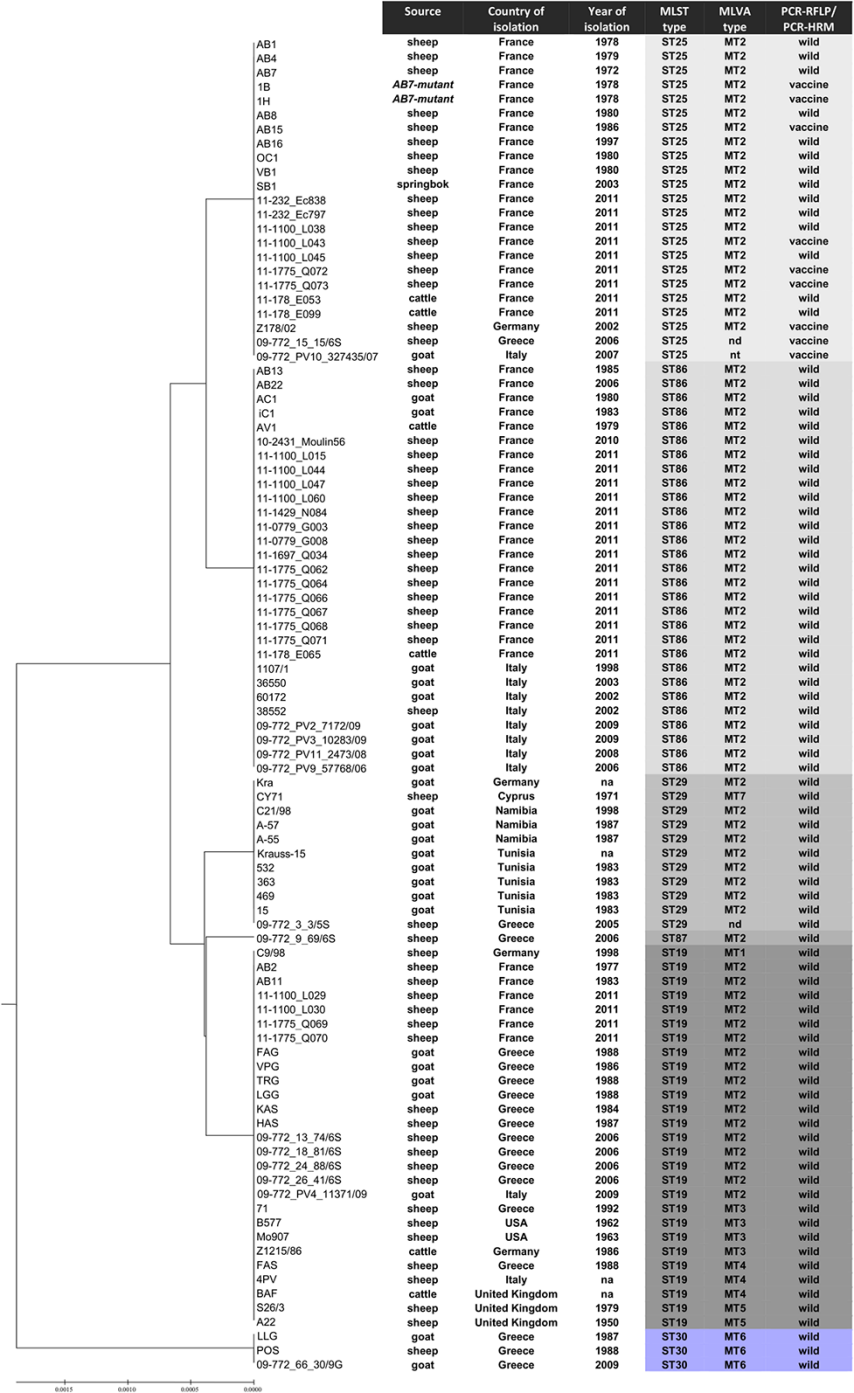
Dendrogram illustrating the relationships of MLST types (STs) on the basis of concatenated sequences of *gatA*, *hemN* and *fumC* loci, in comparison with other genotyping findings. The dendrogram includes 94 *C*. *abortus* strains and field isolates or samples, which were collected from ruminant in nine countries over a period of 60 years. The genotypic characteristics of clones are color-coded: the more distantly related ST30 is presented in blue whereas the other STs in shades of gray. The dendrogram was constructed by using the UPGMA algorithm. Linkage distances are indicated on the scale at the bottom. na, not available information; nd, not determined; nt, not typeable.

In order to study relationships among strains, isolates and samples with particular STs and compare them with the other genotyping findings, a UPGMA dendrogram was constructed based on the concatenated sequences of *gatA*, *hemN* and *fumC* loci of all 94 *C*. *abortus* isolates tested (Fig 1). Two deep branches were evident. The major branch included two distinct clades, one consisting of genotypes ST25 and ST86 that harboured exclusively MT2 with the vaccine-type isolates restricted into ST25, and the other clade consisting of genotypes ST29, ST87 and ST19 that comprised six MTs (MT1 to MT5 and MT7). The more distantly related branch consisted of three *C*. *abortus* isolates exhibiting genotypes ST30 and MT6 exclusively. These differentiated branches or clades might reflect pathways; however, it should be noted that, although clustering algorithms such as UPGMA identify closely related genotypes, they do not provide information on the founding genotypes or the likely patterns of evolutionary descent within a group.

### Identification and delineation of the *C*. *abortus* clonal complex and lineages

**Fig 2:**
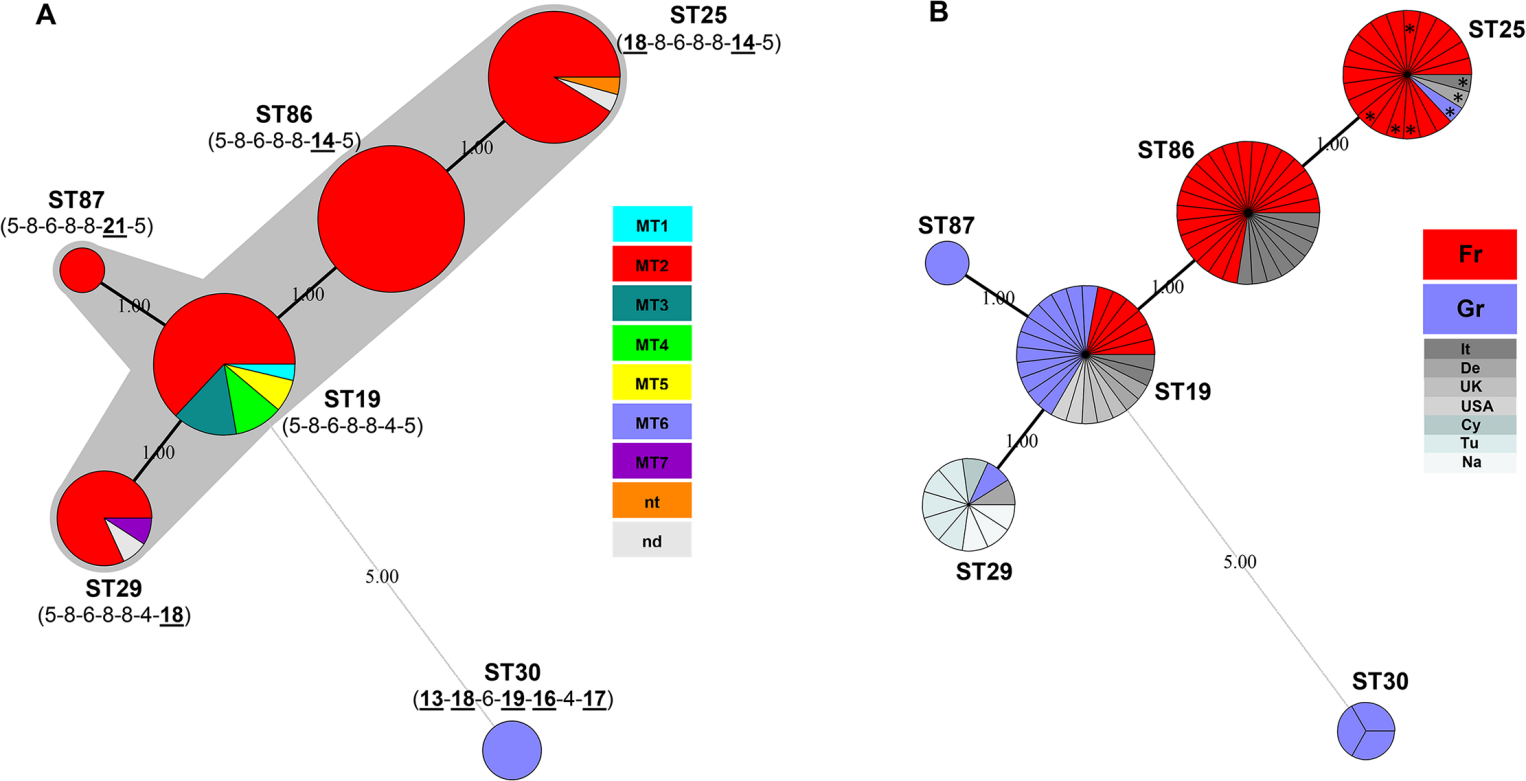
Minimum-spanning-tree analysis of 94 *C*. *abortus* isolates genotyped by MLST. Each circle represents a distinct MLST type (ST). The size of the circles is related to the number of strains, isolates or samples composing the particular STs. Solid lines connect single-locus variant STs while the light grey dashed line indicates the ST that differs in more than one locus; the number of locus variations is indicated between the circles. **A. Minimum-spanning-tree illustrating the evolutionary pathways of ruminant *C*. *abortus* clones.** The gray halo surrounding the circles delineates the *C*. *abortus* clonal complex. Alteration in MLST alleles compared with predicted ancestor ST19 are underlined. The circle colours indicate the corresponding MLVA types (MTs); nd, MT not determined; nt, MT not typeable. **B. Minimum-spanning-tree exemplifying the different ST distribution patterns among *C*. *abortus* strains, isolates and samples originating from France and Greece.** The colours indicate the corresponding countries of isolation (France, red; Greece, blue; Other countries, shades of gray). With asterisks (*) are labeled the field isolates or samples displaying the 1B-vaccine-type profile.

The evolutionary pathways within *C*. *abortus* were reconstructed using minimum-spanning-tree analysis [27]. The analysis of the MLST data of the 94 *C*. *abortus* isolates assigned ST19 as a predicted ancestor. This ST is predicted to have diversified into the single-locus variants ST86, ST87 and ST29, with ST86 to have diversified further to produce its single-locus variant ST25 (double-locus variant of ST19) (Fig 2A). Minimum-spanning-tree analysis suggested that all STs identified in this study, with the exception of ST30, belonged to one clonal complex (Fig 2A). The simple structure of this clonal complex possibly reflects the very short evolutionary timescale over which ST19 has presumably diverged. ST30 was assigned as singleton clone since this ST differed from every other in the dataset at five or six of the seven MLST loci (Fig 2A) and was therefore considered to be distantly related to the clonal complex [29].

Examination of the sequence changes during clonal diversification showed that the single-locus variants possessed variant alleles that were both unique within the dataset (i.e. found only within the single-locus variant) and differed at only a single nucleotide site from the allele in the putative ancestral ST (S2 Fig), characteristics that are consistent with a predominantly mutation mode of evolution [29,34]. Out of the four point mutations that occurred within the clonal complex, three were synonymous and one was nonsynonymous (S2 Fig; listed in S1 Table). It might be expected that synonymous mutations, which are more likely to be neutral, should outweigh nonsynonymous substitutions, since the "core" genes used for MLST are subjected to stabilizing selection. A trend in the direction of positive selection was observed for the nonsynonymous mutation harboured on the novel *hemN*_21 allele (S2 Table).

Minimum-spanning-tree analysis of the ruminant *C*. *abortus* STs and the avian ST36 previously designated as *C*. *abortus* [22], showed the latter presenting a separate singleton topology (S3A Fig). In order to establish the relationships between the *C*. *abortus* clonal complex and other chlamydial clonal lineages, especially the ruminant ST30 and avian ST36 singletons, the degree of phylogenetic consistency between MLST locus trees was examined (S4 Fig). In all trees there was evidence for a conserved *C*. *abortus* branch and conserved division between the *C*. *abortus* clonal complex and the singleton lineages (S4 Fig). Some exceptions to this division, particularly concerning the *hemN* and *hflX* loci, have been noted, demonstrating that some recombination has possibly occurred among these lineages. Interestingly, it has been recently revealed that intraspecific homologous recombination likely contributed significantly to the diversification and evolution of *C*. *trachomatis* and *C*. *psittaci* [35,36]. However, shared alleles among the different lineages (S3B Fig) might instead reflect identity by presumably relatively old descent. It is worth noting that based on rRNA phylogeny the ruminant *C*. *abortus* lineages were found to be descendants of an ancestor common with the avian one [20,22]. An evolutionary bottleneck as a consequence of host niche adaptation resulting in genome degradation [18], might have contributed to the extraordinary genetic conservation and the clonal population structure of *C*. *abortus*. An evolutionary bottleneck history has also been proposed for other bacterial pathogens indicating purely clonal evolution, the so-called monomorphic lineages [37,38]. Meanwhile, genome comparison of the original ST30 strain, namely LLG, with S26/3, a reference wild-type strain belonging to the *C*. *abortus* clonal complex, revealed the greatest variations to occur in pseudogene content [18,19], indicating that the ST30 host adaptation and the associated bottleneck possibly occurred independently from those of the *C*. *abortus* clonal complex lineage.

### Distribution of *C*. *abortus* clonal populations

The *C*. *abortus* strains, isolates and samples examined in this study were collected over a period of 60 years from geographically widespread regions; nine countries, France, Greece, Italy, Germany, the United Kingdom, Cyprus, the United States, Tunisia and Namibia, with the majority originating from the first three (Fig 1). Some *C*. *abortus* clones showed geographic specificity in their distribution, such as ST25 (wild-type isolates) in France, and ST87 as well as the singleton ST30 in Greece.

In particular, the ST19 population was clearly dispersed in Europe and is also found in the United States (Fig 1). However, the evolutionary pathways of *C*. *abortus* STs seem to be diverse across geographic distances, as exemplified by the different ST distribution patterns among *C*. *abortus* strains, isolates and samples originating from France and Greece (Figs 1 and 2B). ST19 was predominant among clones recovered in Greece but, interestingly, in France this clone was less frequent than its descendants ST86 and ST25, even when only taking into account isolates from the same year. It is remarkable that the synonymous changes resulting in the emergence of *hemN*_14 and *gatA*_18 alleles of ST86 and ST25, respectively (Fig 2 and S2 Fig **),** might affect the "competitive" balance between isolates. Random genetic drift and neutral evolution associated with synonymous mutations [39,40] seem to be the dominant evolutionary dynamic that allows alleles to reach high frequencies within the French *C*. *abortus* population. Genetic hitchhiking of the alleles with advantageous mutations at other loci [40,41] might potentially play a role in the frequency of derived *C*. *abortus* clones. Additionally, allele migration into or out of a population through animal mobility, may affect the marked change in allele frequencies and the distribution. With regard to this, it should be noted that French *C*. *abortus* clones were collected from major French breeding areas. In any case, the currently circulating *C*. *abortus* clones in France appeared in the country before the introduction, in the middle 90s, of the virulence-attenuated live 1B vaccine. It is also worth noting that in Greece as well as in Italy and Germany, only the vaccine-type of ST25 has been recovered, clearly after the licensing and commercialization of the 1B vaccine in these countries (Figs 1 and 2B).

ST29 isolates were collected from 1971 to 2005 mostly from southern regions, from Greece, Cyprus, Tunisia and Namibia, and also from Germany (Fig 1). It is unclear whether this clone, being a descendant of the ST19 (Fig 2), has emerged locally, or it has spread globally on at least five occasions. The clone ST87 harbouring the nonsynonymous potentially advantageous mutation on *hemN*_21 allele possibly arose recently in Greece (Table 2 and Fig 1) and, might undergo fixation in the local *C*. *abortus* population. However, genetic investigation of isolates collected locally over a long period may help confirm any hypothesis concerning *C*. *abortus* selection and diversity, particularly for some genotypes that appear to be rare or restricted to particular geographic locations. Such a genotype is the singleton clone ST30. The evidence suggests that this *C*. *abortus* lineage has probably emerged in Northern Greece, has not been disseminated far and wide and has been locally endemic for several years. Interestingly, the same genotype has been retrieved two decades after isolation of the original two strains (LLG and POS) of this lineage (Fig 1). Remarkably, in Northern Greece *C*. *abortus* has been found to be highly diverse even across very short distances. The overall diversity in this area may be the result of independent and parallel evolution of locally differentiated subpopulations that may selectively maintain their favorable genetic background for decades (Fig 1). However, further studies are needed to determine the population dynamics of *C*. *abortus*, both over time and a wider geographic area.

### Combination of MLST and MLVA profiles

Whereas the ST86 and ST25 populations showed the same MLVA profile (MT2) over time and/or geographical distribution, the population of ST19 and, to a lesser extent ST29, diversified into multiple MT subpopulations (Figs 1 and 2A).

As the markers used for MLST (targeting housekeeping genes) evolve slowly and are highly conserved, the resolution provided by MLST is low for the investigation of recent evolution and, above all, for short-term epidemiological studies. Unlike MLST, MLVA targets several types of markers with variation in the numbers of repeats at particular loci, to be used by some bacteria as a means of genomic and phenotypic adaptation to the environment [42]. In our case, three of the VNTR markers that were located within genes or open reading frames encoding predicted membrane or exported proteins (markers ChlaAb_300, ChlaAb_581 and ChlaAb_620/pseudogene) [21] and as surface-located factors presumably could be under selective pressure [43]. Indeed, most of the MT variations observed in the ST19 were due to tandem-repeat differences at either Chla_581 or Chla_620 (mostly) (S5 Fig). Finally, it is important to keep in mind that in the case of MLVA (like in other methods) some similarities in the genotyping patterns might have arisen by convergent evolution [44]. Results presented here indicate that MLVA improved the resolution and discriminated further the ST19 and ST29 subpopulations, possibly enhancing micro-epidemiological accuracy crucial for the understanding of the dissemination of *C*. *abortus* populations in defined areas (Fig 1). Meanwhile, the use of MLST as a backbone to support or further differentiate the MLVA genotypes will be useful in inferring the genetic relationships of *C*. *abortus* lineages and clones. In all strains, we found six distinct STs, seven distinct MTs, and 11 distinct combinations of ST and MT, suggesting that a combination of MLST and MLVA provides additional resolution and may prove useful for the investigation and surveillance of emergent *C*. *abortus* clonal populations.

### Conclusions

In this study the molecular characterization of 94 ruminant *C*. *abortus* strains, isolates and field samples collected from 1950 to 2011 from diverse geographic locations reveals new insights into the population structure, genetic diversity and potentially the evolutionary history of *C*. *abortus*. Taken together, MLST and MLVA analyses demonstrated that *C*. *abortus*, similarly to other genetically monomorphic bacteria, has a predominantly clonal population structure consisting of subpopulations, many of which seem to be associated with particular geographic regions. The analyses further revealed that ruminant *C*. *abortus* strains are more genetically diverse than generally recognized, since novel genotypes have been detected. Both techniques effectively distinguished the "LLG/POS variant" lineage from the "clonal complex" one, supporting the notion that possibly two separate host niche adaptations of *C*. *abortus* have occurred through time. In combination, MLST and MLVA may provide additional information into the origins and evolutionary relationships of circulating *C*. *abortus* populations.

## Supporting Information

S1 Fig VNTR amplicon sizes for the marker ChlaAb_457. PCR amplification of *C*. *abortus* strains AB7 (A), S26/3 (B), POS (C) and CY71 (D). A 100-bp ladder (100–1000 bp) is run on both sides of sample group. The number of repeat units within each allele is indicated.
(TIFF)

S2 Fig Nucleotide substitutions among *C*. *abortus* MLST alleles and comparison with other chlamydial locus alleles (a) Consensus sequence is shown in the first line of the allele sequence alignments generated with BioNumerics software. Consensus blocks are shaded light-gray; 20% has been taking as a minimal fraction of a specific nucleotide at a defined consensus position. **Amino-acid substitutions among *C*. *abortus* MLST-encoded protein alleles (b)** Conserved sites are shaded gray in the protein sequence alignments. **A. Nucleotide substitutions among three *C*. *abortus hemN* alleles (in boldface; *hemN*_4, *hemN*_21, *hemN*_14 ruminant and avian *C*. *abortus* alleles) (a)** The positions in which the *C*. *abortus hemN* alleles present substitutions (positions 261 & 343) are shaded by colours. The substitution (point mutation) that occurs within the clonal complex at the position 261 (alleles *hemN*_4 and *hemN*_14) is synonymous, whereas the substitution at the position 343 (alleles *hemN*_4 and *hemN*_21) is non-synonymous (see b). Residues G and A, at the positions 261 & 343, respectively, are unique among all chlamydial *hemN* alleles. **Amino-acid substitutions among three *C*. *abortus hemN*-encoded protein alleles (b)** The non-synonymous amino-acid Thr (T) harboured at the position 115 of *hemN*_21-encoded protein is unique among all chlamydiae; shaded by distinct colour. **B. Nucleotide substitutions among three *C*. *abortus gatA* alleles (in boldface; *gatA*_5, *gatA*_18, *gatA*_13 ruminant and avian *C*. *abortus* alleles) (a)** The positions in which the *C*. *abortus gatA* alleles present substitutions (positions 165 & 374) are shaded by colours. The substitution (point mutation) that occurs at the position 165 within the clonal complex (alleles *gatA*_5 and *gatA*_18) is synonymous (see b). Residue G at the position 374 characteristic for *C*. *abortus* clonal complex, is unique among all chlamydial *gatA* alleles. **Amino-acid substitutions among three *C*. *abortus gatA*-encoded protein alleles (b)** The amino-acid Gly (G) characterizing the *C*. *abortus* clonal complex is unique among all chlamydial *gatA*-encoded protein alleles at the conserved position 125; shaded by distinct colour. **C. Nucleotide substitutions among four *C*. *abortus fumC* alleles (in boldface; *fumC*_5, *fumC*_18, *fumC*_17, *fumC*_14 ruminant or avian *C*. *abortus* alleles) (a)** The position in which the *C*. *abortus fumC* alleles present substitutions (positions 165, 195, 243 & 440) are shaded by colours. The substitution (point mutation) that occurs at the position 440 within the clonal complex (alleles *fumC*_5 and *fumC*_18) is synonymous (see b). Residue T at position 243 of *fumC_*17 harbouring in the LLG singleton is unique among all chlamydial *fumC* alleles. **Sequence alignment among *C*. *abortus fumC*-encoded protein alleles (b)** Non-synonymous substitutions were not observed. **D. Nucleotide substitutions among three *C*. *abortus enoA* alleles (in boldface; *enoA*_8, *enoA*_16, *enoA*_14 ruminant or avian *C*. *abortus* alleles) (a)** The position in which the *C*. *abortus enoA* alleles present substitutions (positions 249, 291 & 317) are shaded by colours. Residue A at the conserved position 317, is unique among all chlamydial *enoA* alleles. **Amino-acid substitutions among *enoA*-encoded protein alleles (b)** The non-synonymous amino-acid Glu (E) characterizing the LLG singleton is unique among all chlamydial *enoA*-encoded protein alleles at the conserved position 106; shaded by distinct colour.
(PDF)

S3 Fig Minimum-spanning-tree analysis and MLST allelic profiles of ruminant and avian *C*. *abortus* clones. **A.** Minimum-spanning-tree analysis of the ruminant *C*. *abortus* STs and the avian ST36 previously designated as *C*. *abortus* [22]. The gray halo surrounding the circles delineates the *C*. *abortus* clonal complex. The numbers between the circles define the number of locus variations. The circle colours indicate the corresponding MLVA types (MTs); nt, MT not typeable. **B.** The individual MLST allelic profile of ST36 is shown in comparison with the other *C*. *abortus* profiles.
(TIFF)

S4 Fig Maximum likelihood trees. **A.** Maximum likelihood trees are shown for each MLST locus; in each tree locus-alleles corresponding to known chlamydial STs are included. **B.** A maximum likelihood tree, highlighted in red border, is shown on the basis of concatenated sequences of all seven loci corresponding to six ruminant (ST19, ST29, ST87, ST86, ST25, ST30) and one avian (ST36) *C*. *abortus* STs. STs belonging to the clonal complex or singleton lineages are highlighted white on a black or grey background, respectively.
(TIFF)

S5 Fig Minimum-spanning-tree analysis based on MLVA types (MTs). **A.** In the minimum-spanning-tree the MTs are displayed as circles. Circle sizes represent the number of *C*. *abortus* strains, isolates or samples; circles are colored by the corresponding STs (see Fig 1; nt, MT not typeable). Numbers between the circles define the number of locus variations. Thick lines connect MTs that differ in a single VNTR locus while the thin line connects MTs that differ in more than one locus. **B.** The individual MLVA allelic profiles are shown for comparison.
(TIFF)

S1 Table Variant alleles of the single-locus variants found within *C*. *abortus* clonal complex and comparison with LLG singleton clone (ST30). (PDF)

S2 Table Codon-based test of neutrality (a) and positive (b) or purifying (c) selection among *hemN*-locus sequences. (PDF)
