# Supplementary figures and images for: Diversification and Distribution of Ruminant Chlamydia abortus Clones Assessed by MLST and MLVA
Source: PLoS One. 2015 May 22;10(5):e0126433. doi: 10.1371/journal.pone.0126433 (PMC4441495; doi:10.1371/journal.pone.0126433)

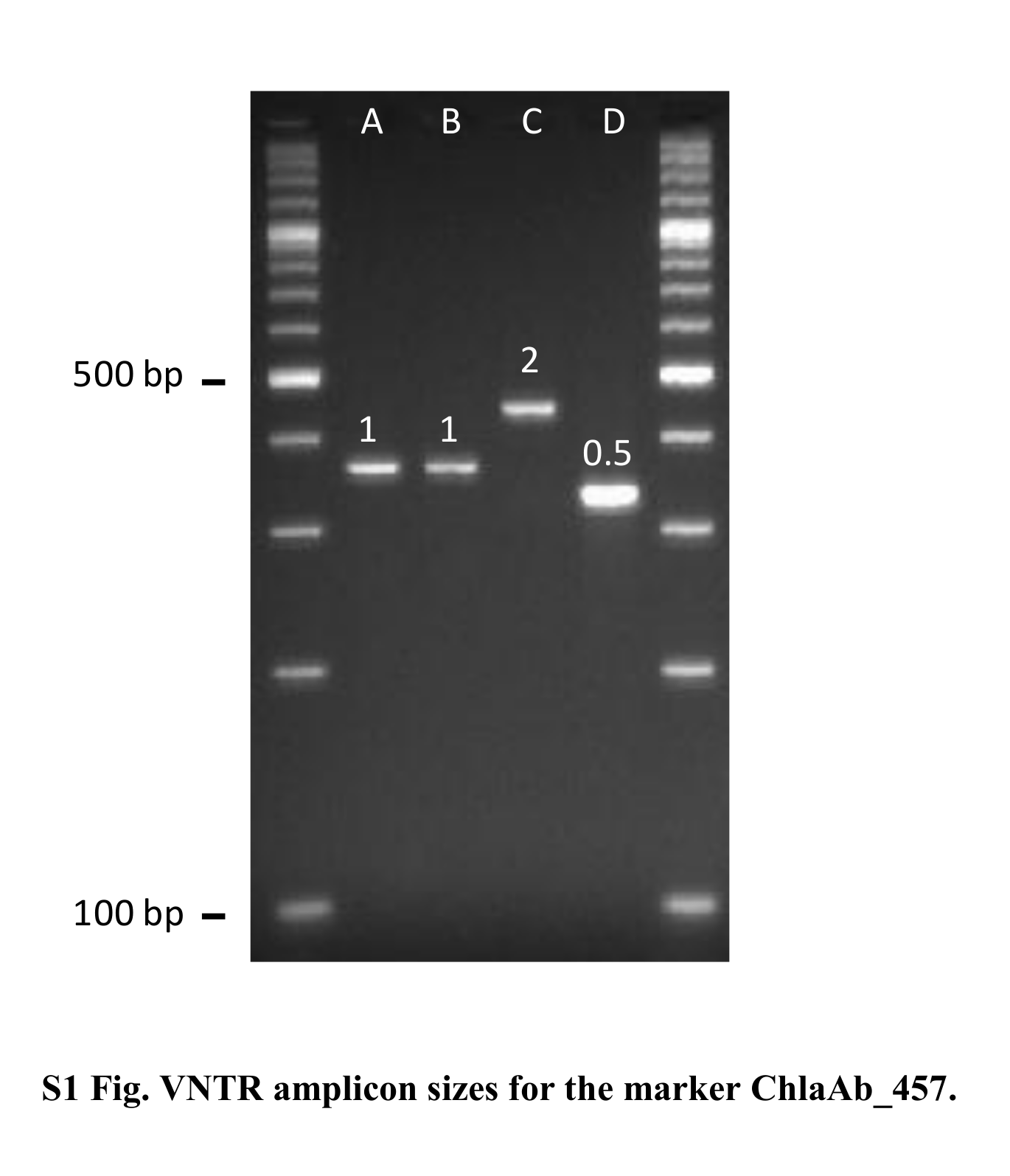

Supplement: S1 Fig — PCR amplification of C. abortus strains AB7 (A), S26/3 (B), POS (C) and CY71 (D). A 100-bp ladder (100–1000 bp) is run on both sides of sample group. The number of repeat units within each allele is indicated. (TIFF) [file pone.0126433.s001.tiff]

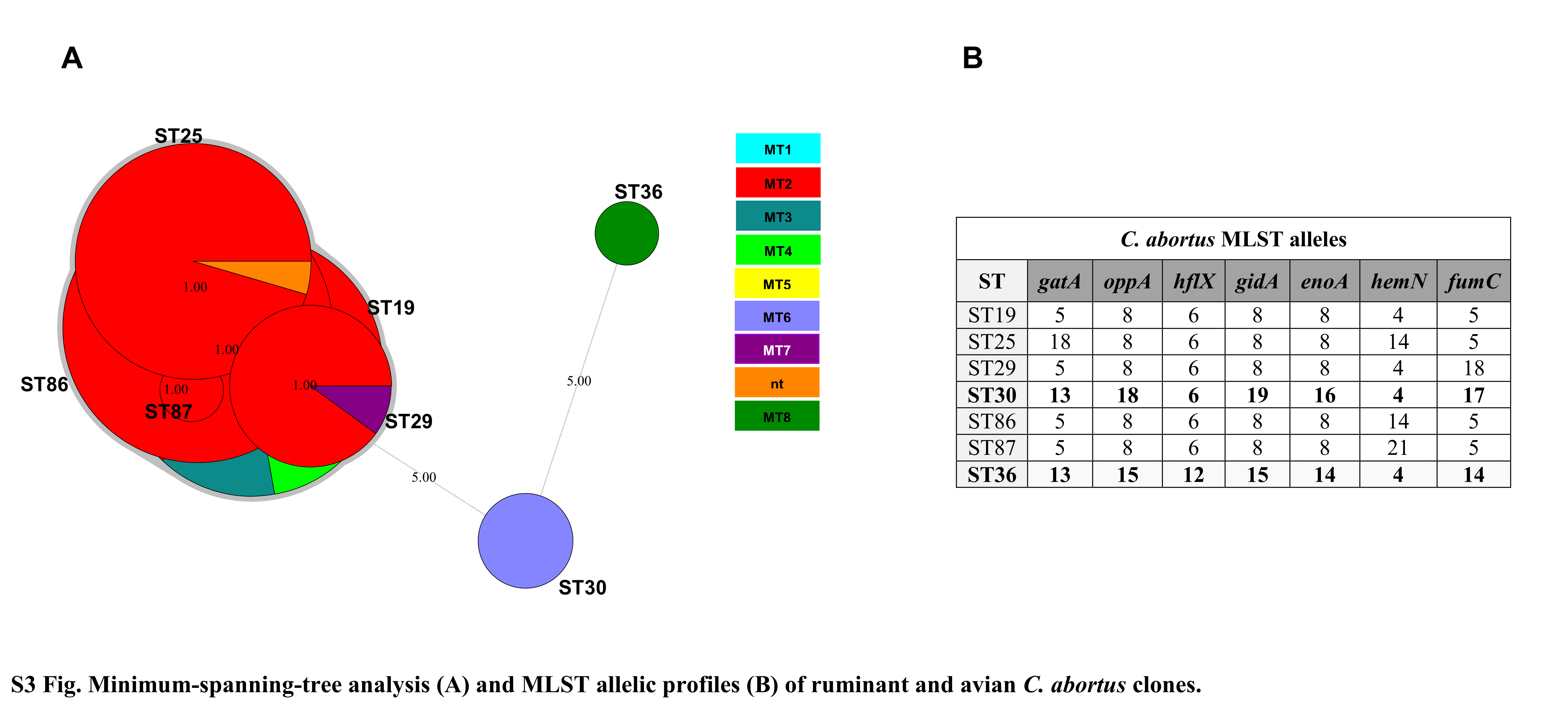

Supplement: S3 Fig — A. Minimum-spanning-tree analysis of the ruminant C. abortus STs and the avian ST36 previously designated as C. abortus [22]. The gray halo surrounding the circles delineates the C. abortus clonal complex. The numbers between the circles define the number of locus variations. The circle colours indicate the corresponding MLVA types (MTs); nt, MT not typeable. B. The individual MLST allelic profile of ST36 is shown in comparison with the other C. abortus profiles. (TIFF) [file pone.0126433.s003.tiff]

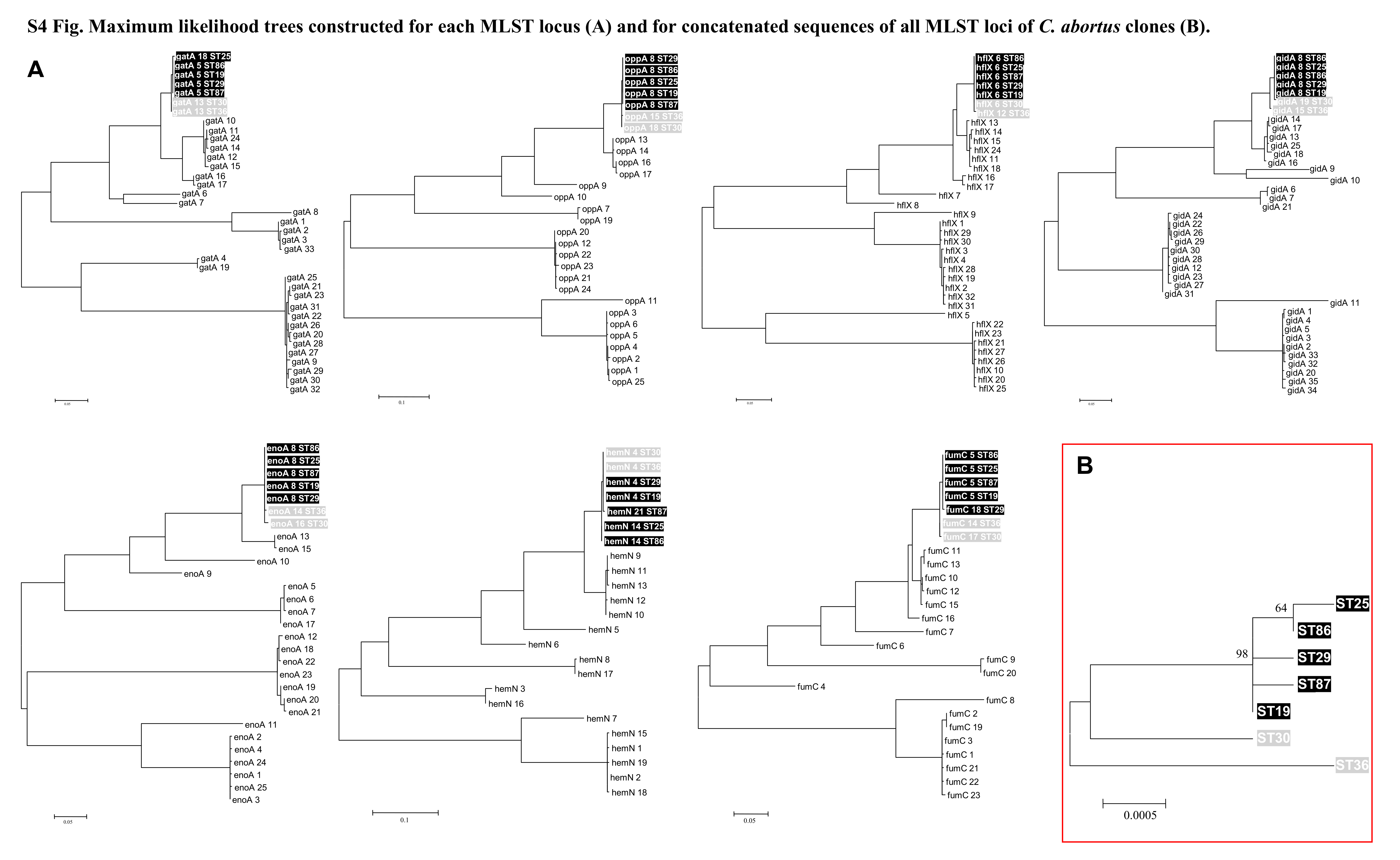

Supplement: S4 Fig — A. Maximum likelihood trees are shown for each MLST locus; in each tree locus-alleles corresponding to known chlamydial STs are included. B. A maximum likelihood tree, highlighted in red border, is shown on the basis of concatenated sequences of all seven loci corresponding to six ruminant (ST19, ST29, ST87, ST86, ST25, ST30) and one avian (ST36) C. abortus STs. STs belonging to the clonal complex or singleton lineages are highlighted white on a black or grey background, respectively. (TIFF) [file pone.0126433.s004.tiff]

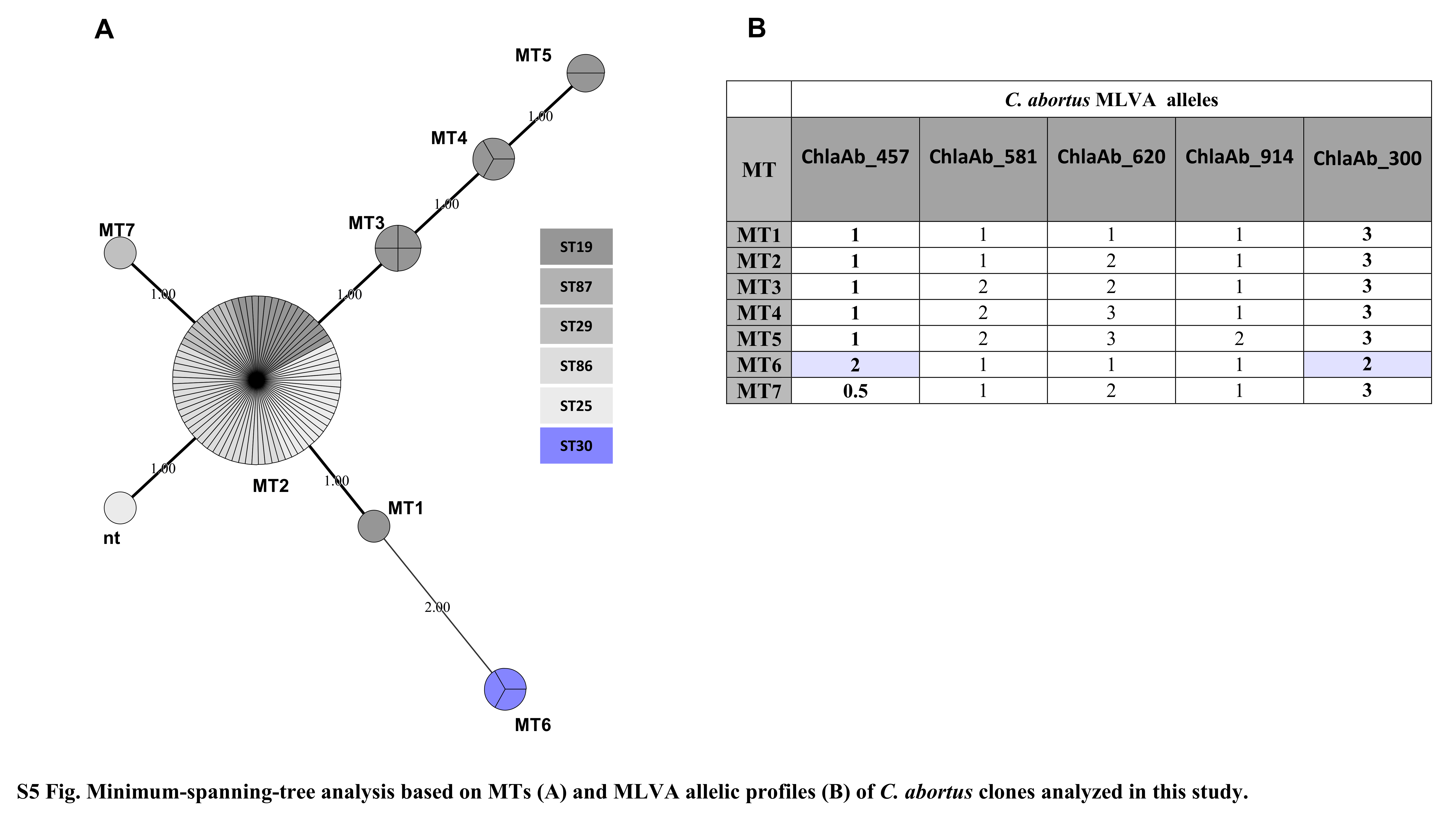

Supplement: S5 Fig — A. In the minimum-spanning-tree the MTs are displayed as circles. Circle sizes represent the number of C. abortus strains, isolates or samples; circles are colored by the corresponding STs (see Fig 1; nt, MT not typeable). Numbers between the circles define the number of locus variations. Thick lines connect MTs that differ in a single VNTR locus while the thin line connects MTs that differ in more than one locus. B. The individual MLVA allelic profiles are shown for comparison. (TIFF) [file pone.0126433.s005.tiff]
