# Supplementary material for: Diversification and Distribution of Ruminant Chlamydia abortus Clones Assessed by MLST and MLVA
Source: PLoS One. 2015 May 22;10(5):e0126433. doi: 10.1371/journal.pone.0126433 (PMC4441495; doi:10.1371/journal.pone.0126433)
Supplement: S2 Fig — Consensus blocks are shaded light-gray; 20% has been taking as a minimal fraction of a specific nucleotide at a defined consensus position. Amino-acid substitutions among C. abortus MLST-encoded protein alleles (b) Conserved sites are shaded gray in the protein sequence alignments. A. Nucleotide substitutions among three C. abortus hemN alleles (in boldface; hemN_4, hemN_21, hemN_14 ruminant and avian C. abortus alleles) (a) The positions in which the C. abortus hemN alleles present substitutions (positions 261 & 343) are shaded by colours. The substitution (point mutation) that occurs within the clonal complex at the position 261 (alleles hemN_4 and hemN_14) is synonymous, whereas the substitution at the position 343 (alleles hemN_4 and hemN_21) is non-synonymous (see b). Residues G and A, at the positions 261 & 343, respectively, are unique among all chlamydial hemN alleles. Amino-acid substitutions among three C. abortus hemN-encoded protein alleles (b) The non-synonymous amino-acid Thr (T) harboured at the position 115 of hemN_21-encoded protein is unique among all chlamydiae; shaded by distinct colour. B. Nucleotide substitutions among three C. abortus gatA alleles (in boldface; gatA_5, gatA_18, gatA_13 ruminant and avian C. abortus alleles) (a) The positions in which the C. abortus gatA alleles present substitutions (positions 165 & 374) are shaded by colours. The substitution (point mutation) that occurs at the position 165 within the clonal complex (alleles gatA_5 and gatA_18) is synonymous (see b). Residue G at the position 374 characteristic for C. abortus clonal complex, is unique among all chlamydial gatA alleles. Amino-acid substitutions among three C. abortus gatA-encoded protein alleles (b) The amino-acid Gly (G) characterizing the C. abortus clonal complex is unique among all chlamydial gatA-encoded protein alleles at the conserved position 125; shaded by distinct colour. C. Nucleotide substitutions among four C. abortus fumC alleles (in boldface; fu [file pone.0126433.s002.pdf]

S2 Fig.

A. Nucleotide substitutions among three *C. abortus hemN* alleles (in boldface; *hemN*_4, *hemN*_21, *hemN*_14 ruminant and avian *C. abortus* alleles) (a)

```
                   .10       .20       .30       .40       .50       .60       .70       .80       .90       .100      .110      .120      .130      .140      .150      .160      .170      .180      .190      .200      .210
consensus CGTGTGAGYYTRGGHRTYCARGATACCCAAKSKGMWGTTCAAGAAGCTGTWCGKMGWCGBCAAWSTYAYGARGARTCDCTNYWWGCTTATSAAMWATTYMRWGASYTARRWTTYRMARGYATHAAYATWGAYYTMATTTAYGGTCTYCCWAARCAAACNMAGKHWASHTTTAMDCAAACVATTSMWSATATYTTASMBATGCGKCCWGAY
hemN_4    CGTGTGAGCTTAGGCGTCCAAGATACCCAAGCTGCTGTTCAAGAAGCTGTACGGCGTCGGCAAAGTTACGAAGAGTCACTGTATGCTTATGAAAAATTCCGTGAGTTAGGTTTTGAAAGCATAAATATAGATCTCATTTACGGTCTTCCAAAACAAACGAAGGTAACATTTACGCAAACGATTGCGGATATTTTACACATGCGTCCTGAC
hemN_21   CGTGTGAGCTTAGGCGTCCAAGATACCCAAGCTGCTGTTCAAGAAGCTGTACGGCGTCGGCAAAGTTACGAAGAGTCACTGTATGCTTATGAAAAATTCCGTGAGTTAGGTTTTGAAAGCATAAATATAGATCTCATTTACGGTCTTCCAAAACAAACGAAGGTAACATTTACGCAAACGATTGCGGATATTTTACACATGCGTCCTGAC
hemN_14   CGTGTGAGCTTAGGCGTCCAAGATACCCAAGCTGCTGTTCAAGAAGCTGTACGGCGTCGGCAAAGTTACGAAGAGTCACTGTATGCTTATGAAAAATTCCGTGAGTTAGGTTTTGAAAGCATAAATATAGATCTCATTTACGGTCTTCCAAAACAAACGAAGGTAACATTTACGCAAACGATTGCGGATATTTTACACATGCGTCCTGAC
hemN_9    CGTGTGAGCTTAGGAGTCCAAGATACCCAAGCTGCTGTTCAAGAAGCTGTACGGCGTCGTCAAAGTCATGAAGAGTCACTTTATGCTTATGAAAAATTCCGTGAGTTAGGTTTTGAAAGCATAAATATAGATCTAATTTACGGTCTTCCAAAACAAACTAAGTTAACATTTACTCAAACGATTGCTGATATTTTACACATGCGGCCTGAC
hemN_11   CGTGTGAGCTTAGGAGTCCAAGATACCCAAGCTGCTGTTCAAGAAGCTGTACGGCGTCGTCAAAGTCATGAAGAGTCACTTTATGCTTATGAAAAATTCCGTGAGTTAGGTTTTGAAAGCATAAATATAGATCTAATTTACGGTCTTCCAAAACAAACTAAGTTAACATTTACTCAAACGATTGCTGATATTTTACACATGCGGCCTGAC
hemN_13   CGTGTGAGCTTAGGAGTCCAAGATACCCAAGCTGCTGTTCAAGAAGCTGTACGGCGTCGTCAAAGTCATGAAGAGTCACTTTATGCTTATGAAAAATTCCGTGAGTTAGGTTTTGAAAGCATAAATATAGATCTAATTTACGGTCTTCCAAAACAAACTAAGTTAACATTTACTCAAACGATTGCTGATATTTTACACATGCGGCCTGAC
hemN_10   CGTGTGAGCTTAGGAGTCCAAGATACCCAAGCTGCTGTTCAAGAAGCTGTACGGCGTCGTCAAAGTCATGAAGAGTCACTTTATGCTTATGAAAAATTCCGTGAGTTAGGTTTTGAAAGCATAAATATAGATCTAATTTACGGTCTTCCAAAACAAACTAAGTTAACATTTACTCAAACGATTGCTGATATTTTACACATGCGGCCTGAC
hemN_12   CGTGTGAGCTTAGGAGTCCAAGATACCCAAGCTGCTGTTCAAGAAGCTGTACGGCGTCGTCAAAGTCATGAAGAGTCACTTTATGCTTATGAAAAATTCCGTGAGTTAGGTTTTGAAAGCATAAATATAGATCTAATTTACGGTCTTCCAAAACAAACTAAGTTAACATTTACTCAAACGATTGCTGATATTTTACACATGCGGCCTAAC
hemN_5    CGTGTGAGCTTAGGGGTTCAAGATACGCAAGCTGCTGTTCAAGAAGCTGTACGGCGACGACAAAGTCATGAAGAATCTCTGCATGCTTATGAGAAGTTTCGTGCTCTAGGTTTTGAGAGTATAAACATTGATTTGATTTATGGTCTCCCCAAGCAAACAAAAGCAACATTTACCCAAACAATCGCTGATATTTTACAGATGCGTCCTGAC
hemN_6    CGCGTGAGCCTAGGAGTACAGGATACCCAAGCTTCTGTTCAAGAGGCTGTGCGGCGGCGTCAAAGTTATGAAGAGTCTCTACATGCCTATGAAAAATTTCGAGAGCTAGGTTTTGAGAGTATAAACATAGATTTGATTTACGGACTTCCTAAGCAAACAAAGAAAACATTTTCACAAACAATTGTAGATATTTTACAGATGCGTCCCGAT
hemN_8    CGTGTAAGCTTGGGAGTACAAGATACCCAAGCTGCCGTACAAGAAGCCGTAAAGCGACGGCAGACCCATGAGGAGTCACGACGTGTGTATGAGAAGTTTAAAGAGCTGGGCTTTACTAGCATCAACATTGATCTAATCTACGGCTTGCCAAAGCAAACCCGTCACACCTTTGCGCAAACTATCCAAGACATCCTCGCAATGCGCCCGGAT
hemN_17   CGTGTAAGCTTGGGAGTACAAGATACCCAAGCTGCCGTACAAGAAGCCGTAAAGCGACGGCAGACCCATGAGGAGTCACGACGTGTGTATGAGAAGTTTAAAGAGCTGGGTTTTACTAGCATCAACATTGATCTAATCTACGGCTTGCCAAAGCAAACCCGTCACACCTTTGCGCAAACTATCCAAGACATCCTCGCAATGCGCCCGGAT
hemN_3    AGGGTTAGCTTAGGCGTTCAAGATACCCAAGCTGATGTTCAAGAAGCTGTACGACGACGCCAATCGCATGAGGAATCTTTAAAGGCATACGAAAAATTTAAGGAACTCGCCTTCCAAAGTATCAATATAGACTTAATTTATGGTCTTCCCAAACAAACCAAGGAGTCCTTTTCTAAAACAATTCAAGATATCTTAGCGATGTATCCAGAT
hemN_16   AGGGTTAGCTTAGGCGTTCAAGATACCCAAGCTGATGTTCAAGAAGCTGTACGACGACGCCAATCGCATGAGGAATCTTTAAAGGCATACGAAAAATTTAAGGAACTCGGCTTCCAAAGTATCAATATAGACTTAATTTATGGTCTTCCCAAGCAAACCAAGGAGTCCTTTTCTAAAACAATTCAAGATATCTTAGCGATGTATCCAGAT
hemN_7    CGTGTCAGCCTTGGAATCCAAGATACTCAATGGGAAGTTCAAGAGGCCGTCCGTAGACGCCAATCCTATGAAGAATCCCTCTTGGCCTATAAACAATTCCGTGATCTGAATTTCACAGGTATCAATATTGATCTTATTTATGGGCTTCCTAAACAAACGCAATCGAGTTTCAAACAAACCATTCAACATATTTTAGATATGCGGCCTGAT
hemN_15   CGTGTGAGTCTGGGTATTCAGGATACCCAATGGGAAGTTCAAGAAGCTGTTCGTAGACGCCAATCTTACGAAGAATCGCTCTTAGCTTATCAACTATTCCGAGACTTAAAATTCACAGGTATTAATATTGACCTCATTTATGGTCTCCCTAAACAAACACAGTCTAGTTTTAAACAAACCATTGAACATATCTTAGATATGCGGCCAGAC
hemN_19   CGTGTGAGTCTGGGTATTCAGGATACCCAATGGGAAGTTCAAGAAGCTGTTCGTAGACGCCAATCTTACGAAGAATCGCTCTTAGCTTATCAACTATTCCGAGACTTAAAATTCACAGGTATTAATATTGACCTCATTTACGGTCTCCCTAAACAAACACAGTCTAGTTTTAAACAAACCATTGAACATATCTTAGATATGCGGCCAGAC
hemN_1    CGTGTGAGTCTGGGTATTCAGGATACCCAATGGGAAGTTCAAGAAGCTGTTCGTAGACGCCAATCTTACGAAGAATCGCTCTTAGCTTATCAACTATTCCGAGACTTAAAATTCACAGGTATTAATATTGACCTCATTTATGGTCTCCCTAAACAAACACAGTCTAGTTTTAAACAAACCATTGAACATATCTTAGATATGCGGCCAGAC
hemN_2    CGTGTGAGTCTGGGTATTCAGGATACCCAATGGGAAGTTCAAGAAGCTGTTCGTAGACGCCAATCTTACGAAGAATCGCTCTTAGCTTATCAACTATTCCGAGACTTAAAATTCACAGGTATTAATATTGACCTCATTTATGGTCTCCCTAAACAAACACAGTCTAGTTTTAAACAAACCATTGAACATATCTTAGATATGCGGCCAGAC
hemN_18   CGTGTGAGTCTGGGTATTCAGGATACCCAATGGGAAGTTCAAGAAGCTGTTCGTAGACGCCAATCTTACGAAGAATCGCTCTTAGCTTATCAACTATTCCGAGACTTAAAATTCACAGGTATTAATATTGACCTCATTTATGGTCTCCCTAAACAAACACAGTCTAGTTTTAAACAAACCATTGAACATATCTTAGATATGCGGCCAGAC
                   .220      .230      .240      .250      .260      .270      .280      .290      .300      .310      .320      .330      .340      .350      .360      .370      .380      .390      .400      .410      .420
consensus CGYYTAGCSYTWTTYTCHTTYGCTYMWGTWCCWTGGRYHAARCCKCAYCAAAARGCTWTGMRMRMWAVHGAYMTSCCYTCYATGGARGARAAATTCGCSATWTATTCYYAWKCYCGRCATACSYTRAYVMARGMAGGHTAYCARGCYATTGGWHTRGATCATTTTTCTCTWCCBGAWGAYCCWYTAASYATMGCBYTTAARAACAAAACK
hemN_4    CGCTTAGCGTTATTTTCATTTGCTTCTGTTCCCTGGATAAAACCCCATCAGAAAGCTATGAAAGAAAGTGACATGCCCTCTATGGAGGAAAAATTCGCCATATATTCTTATGCTCGGCATACGTTAACAAAAGCTGGTTACCAAGCTATTGGATTAGATCATTTTTCTCTACCTGAAGACCCATTAAGCATCGCCTTTAAAAACAAAACT
hemN_21   CGCTTAGCGTTATTTTCATTTGCTTCTGTTCCCTGGATAAAACCCCATCAGAAAGCTATGAAAGAAAGTGACATGCCCTCTATGGAGGAAAAATTCGCCATATATTCTTATGCTCGGCATACGTTAACAAAAACTGGTTACCAAGCTATTGGATTAGATCATTTTTCTCTACCTGAAGACCCATTAAGCATCGCCTTTAAAAACAAAACT
hemN_14   CGCTTAGCGTTATTTTCATTTGCTTCTGTTCCCTGGATAAAACCCCATCAAAAAGCTATGAAAGAAAGTGACATGCCCTCTATGGAGGAAAAATTCGCCATATATTCTTATGCTCGGCATACGTTAACAAAAGCTGGTTACCAAGCTATTGGATTAGATCATTTTTCTCTACCTGAAGACCCATTAAGCATCGCCTTTAAAAACAAAACT
hemN_9    CGCTTAGCGTTATTTTCATTTGCTTCTGTTCCATGGATAAAACCTCATCAAAAAGCTATGAAAGAAAGCGATATGCCCTCTATGGAGGAAAAATTCGCCATATATTCTTATGCTCGACATACGTTAACGAAAGCAGGTTACCAAGCCATTGGATTAGATCATTTTTCTCTACCTGAAGACCCATTAAGCATAGCGTTTAAAAACAAAACT
hemN_11   CGCTTAGCGTTATTTTCATTTGCTTCTGTTCCATGGATAAAACCTCATCAAAAAGCTATGAAAGAAAGCGATATGCCCTCTATGGAGGAAAAATTCGCCATATATTCTTATGCTCGACATACGTTAACGAAAGCAGGTTACCAAGCCATTGGATTAGATCATTTTTCTCTACCTGAAGACCCATTAAGCATAGCGTTTAAAAACAAAACT
hemN_13   CGCTTAGCGTTATTTTCATTTGCTTCTGTTCCATGGATAAAACCTCATCAAAAAGTTATGAAAGAAAGCGATATGCCCTCTATGGAGGAAAAATTCGCCATATATTCTTATGCTCGACATACGTTAACGAAAGCAGGTTACCAAGCCATTGGATTAGATCATTTTTCTCTACCTGAAGACCCATTAAGCATAGCGTTTAAAAACAAAACT
hemN_10   CGCTTAGCGTTATTTTCATTTGCTTCTGTTCCATGGATAAAACCTCATCAAAAAGCTATGAAAGAAAGCGATATGCCCTCTATGGAGGAAAAATTCGCCATATATTCTTATGCTCGACATACGTTAACGAAAGCAGGTTACCAAGCCATTGGATTAGATCATTTTTCTCTACCTGAAGACCCATTAAGCATAGCGTTTAAAAACAAAACT
hemN_12   CGCTTAGCGTTATTTTCATTTGCTTCTGTTCCATGGATAAAACCTCATCAAAAAGCTATGAAAGAAAGCGATATGCCCTCTATGGAGGAAAAATTCGCCATATATTCTTATGCTCGACATACGTTAACGAAAGCAGGTTACCAAGCCATTGGATTAGATCATTTTTCTCTACCTGAAGACCCATTAAGCATAGCTTTTAAAAACAAAACT
hemN_5    CGCCTAGCGTTGTTTTCATTTGCTTCTGTGCCTTGGATAAAGCCTCATCAAAAAGCTATGAAAGAAAGTGACATGCCCTCCATGGAGGAGAAATTCGCTATATACTCTTATGCACGGCATAAATTAACAAAGTCGGGATATCAGGCAATTGGTTTAGATCATTTTTCACTACCGGAAGATCCTTTAAGCATAGCCTTTAAAAATAAAACT
hemN_6    CGTTTAGCCTTGTTTTCCTTCGCTTCTGTTCCATGGATAAAACCGCACCAAAAAGCAATGAAAGACAGTGATCTGCCCTCTATGGAGGAAAAATTCGCGATATATTCTCATGCACGACATATGTTAACAAAGTCGGGATATCAGGCTATTGGCTTGGATCATTTTTCCCTACCCGAAGACCCTTTAAGCATCGCTTTAAAAAATAAAACT
hemN_8    CGTCTGGCATTATTTTCTTATGCTGCAGTCCCTTGGATCAAACCGCACCAAAAAGCAATCAAAGCTACAGATCTTCCCTCTATGGAAGAAAAATTTGCGATATACTCACAATCCCGACATAGCTTAATCAAGGCAGGCTACCAAGCTATTGGGATGGATCATTTTTCTCTTCCCGAAGATCCCTTAACAGTAGCGTTTCATAACAAAACA
hemN_17   CGTCTGGCATTATTTTCTTATGCTGCAGTCCCTTGGATCAAACCGCACCAAAAAGCAATCAAAGCTACAGATCTTCCCTCTATGGAAGAAAAATTTGCGATATACTCACAATCCCGACATAGCTTAATCAAGGCAGGCTACCAAGCTATTGGGATGGATCATTTTTCTCTTCCCGAAGATCCCTTAACAGTAGCGTTTCATAACAAAACA
hemN_3    CGTCTTGCTTTATTTTCTTTTGCCTCAGTTCCATGGATCAAGCCGCACCAAAAAGCCATGAAAGCTTCGGATATGCCTTCTATGGAAGAGAAATTCGCGATTTATTCTCAATCCCGGCATTTACTTACAAAAGCAGGATATCAGGCCATCGGTATGGATCATTTCTCTCTTCCCCATGATCCTCTTACCCTCGCTTTCAAAAACAAAACT
hemN_16   CGTCTTGCTTTATTTTCTTTTGCCTCAGTTCCATGGATCAAGCCGCACCAAAAAGCCATGAAAGCTTCGGATATGCCTTCTATGGAAGAGAAATTCGCGATTTATTCTCAATCCCGGCATTTACTTACAAAAGCAGGATATCAGGCCATCGGTATGGATCATTTCTCTCTTCCCCATGATCCTCTTACCCTTGCTTTCAAAAACAAAACT
hemN_7    CGCTTAGCCCTATTCTCTTTTGCACATGTGCCATGGGTCAAGCCTCATCAAAAAGCTTTACGAACAAAAGACCTTCCCTCTATGGAAGAGAAATTTGCGATTTATTCTCAATCTCGACAAGCCCTTATCCAAGAAGGATACCAGGCTATTGGCCTTGATCATTTTTCTCTTCCTGATGATCCCCTAACTATTGCACTTAAAAACAAAACC
hemN_15   CGCCTAGCCCTTTTCTCCTTCGCTCATGTACCTTGGACTAAGCCGCATCAAAAGGCTTTGCGCACTAAAGATCTCCCTTCCATGGAAGAGAAATTCGCGATTTATTCCCAATCTCGGCATACCCTGATCCAGGAAGGCTACCAGGCTATTGGTCTAGATCATTTTTCTCTTCCGGATGATCCTCTAACTATCGCCCTTAAGAACAAAACG
hemN_19   CGCCTAGCCCTTTTCTCCTTCGCTCATGTACCTTGGGCTAAGCCGCATCAAAAGGCTTTGCGCACTAAAGATCTCCCTTCCATGGAAGAGAAATTCGCGATTTATTCCCAATCTCGGCATACCCTGATCCAGGAAGGCTACCAGGCTATTGGTCTAGATCATTTTTCTCTTCCGGATGATCCTCTAACTATCGCCCTTAAGAACAAAACG
hemN_1    CGCCTAGCCCTTTTCTCCTTCGCTCATGTACCTTGGGCTAAGCCGCATCAAAAGGCTTTGCGCACTAAAGAGCTCCCTTCCATGGAAGAGAAATTCGCGATTTATTCCCAATCTCGGCATACCCTGATCCAGGAAGGCTACCAGGCTATTGGTCTAGATCATTTTTCTCTTCCGGATGATCCTCTAACTATCGCCCTTAAGAACAAAACG
hemN_2    CGCCTAGCCCTTTTCTCCTTCGCTCATGTACCTTGGGCTAAGCCGCATCAAAAGGCTTTGCGCACTAAAGATCTCCCTTCCATGGAAGAGAAATTCGCGATTTATTCCCAATCTCGGCATACCCTGATCCAGGAAGGCTACCAGGCTATTGGTCTAGATCATTTTTCTCTTCCGGATGATCCTCTAACTATCGCCCTTAAGAACAAAACG
hemN_18   CGCCTAGCCCTTTTCTCCTTCGCTCATGTACCTTGGGCTAAGCCGCATCAAAAGGCTTTGCGCACTAAAGATCTCCCTTCCATGGAAGAGAAATTCGCGATTTATTCCCAATCTCGGCATACCCTGATCCAGGAAGGCTACCAGGCTATTGGTCTAGATCATTTTTCTCTTCCGGATGATCCTCTAACTATCGCCCTTAAGAACAAAACG

                   .430
consensus YTRATTCGDAAT
hemN_4    TTGATTCGAAAT
hemN_21   TTGATTCGAAAT
hemN_14   TTGATTCGAAAT
hemN_9    TTGATTCGGAAT
hemN_11   TTGATTAGGAAT
hemN_13   TTGATTCGGAAT
hemN_10   TTGATTCGGAAT
hemN_12   TTGATTCGGAAT
hemN_5    CTGATTCGTAAT
hemN_6    CTTATTCGCAAT
hemN_8    TTAATTCGTAAT
hemN_17   TTAATTCGTAAT
hemN_3    CTAATCCGCAAC
hemN_16   CTAATCCGTAAC
hemN_7    CTTATTCGAAAT
hemN_15   TTGATTCGTAAT
hemN_19   TTGATTCGTAAT
hemN_1    TTGATTCGTAAT
hemN_2    TTGATTCGTAAT
hemN_18   TTGATTCATAAT
```

A. Amino-acid substitutions among three *C. abortus hemN*-encoded protein alleles (b)

```
                                                                                                               111111111111111111111111111111111111111111111
                   111111111122222222223333333333444444444455555555556666666666777777777788888888889999999999000000000011111111112222222222333333333344444
          123456789012345678901234567890123456789012345678901234567890123456789012345678901234567890123456789012345678901234567890123456789012345678901234
hemN_4    RVSLGVQDTQAAVQEAVRRRQSYEESLYAYEKFRELGFESINIDLIYGLPKQTKVTFTQTIADILHMRPDRLALFSFASVPWIKPHQKAMKESDMPSMEEKFAIYSYARHTLTKAGYQAIGLDHFSLPEDPLSIAFKNKTLIRN
hemN_21   RVSLGVQDTQAAVQEAVRRRQSYEESLYAYEKFRELGFESINIDLIYGLPKQTKVTFTQTIADILHMRPDRLALFSFASVPWIKPHQKAMKESDMPSMEEKFAIYSYARHTLTKTGYQAIGLDHFSLPEDPLSIAFKNKTLIRN
hemN_14   RVSLGVQDTQAAVQEAVRRRQSYEESLYAYEKFRELGFESINIDLIYGLPKQTKVTFTQTIADILHMRPDRLALFSFASVPWIKPHQKAMKESDMPSMEEKFAIYSYARHTLTKAGYQAIGLDHFSLPEDPLSIAFKNKTLIRN
hemN_9    RVSLGVQDTQAAVQEAVRRRQSHEESLYAYEKFRELGFESINIDLIYGLPKQTKLTFTQTIADILHMRPDRLALFSFASVPWIKPHQKAMKESDMPSMEEKFAIYSYARHTLTKAGYQAIGLDHFSLPEDPLSIAFKNKTLIRN
hemN_11   RVSLGVQDTQAAVQEAVRRRQSHEESLYAYEKFRELGFESINIDLIYGLPKQTKLTFTQTIADILHMRPDRLALFSFASVPWIKPHQKAMKESDMPSMEEKFAIYSYARHTLTKAGYQAIGLDHFSLPEDPLSIAFKNKTLIRN
hemN_13   RVSLGVQDTQAAVQEAVRRRQSHEESLYAYEKFRELGFESINIDLIYGLPKQTKLTFTQTIADILHMRPDRLALFSFASVPWIKPHQKVMKESDMPSMEEKFAIYSYARHTLTKAGYQAIGLDHFSLPEDPLSIAFKNKTLIRN
hemN_10   RVSLGVQDTQAAVQEAVRRRQSHEESLYAYEKFRELGFESINIDLIYGLPKQTKLTFTQTIADILHMRPDRLALFSFASVPWIKPHQKAMKESDMPSMEEKFAIYSYARHTLTKAGYQAIGLDHFSLPEDPLSIAFKNKTLIRN
hemN_12   RVSLGVQDTQAAVQEAVRRRQSHEESLYAYEKFRELGFESINIDLIYGLPKQTKLTFTQTIADILHMRPNRLALFSFASVPWIKPHQKAMKESDMPSMEEKFAIYSYARHTLTKAGYQAIGLDHFSLPEDPLSIAFKNKTLIRN
hemN_5    RVSLGVQDTQAAVQEAVRRRQSHEESLHAYEKFRALGFESINIDLIYGLPKQTKATFTQTIADILQMRPDRLALFSFASVPWIKPHQKAMKESDMPSMEEKFAIYSYARHKLTKSGYQAIGLDHFSLPEDPLSIAFKNKTLIRN
hemN_6    RVSLGVQDTQASVQEAVRRRQSYEESLHAYEKFRELGFESINIDLIYGLPKQTKKTFSQTIVDILQMRPDRLALFSFASVPWIKPHQKAMKDSDLPSMEEKFAIYSHARHMLTKSGYQAIGLDHFSLPEDPLSIALKNKTLIRN
hemN_8    RVSLGVQDTQAAVQEAVKRRQTHEESRRVYEKFKELGFTSINIDLIYGLPKQTRHTFAQTIQDILAMRPDRLALFSYAAVPWIKPHQKAIKATDLPSMEEKFAIYSQSRHSLIKAGYQAIGMDHFSLPEDPLTVAFHNKTLIRN
hemN_17   RVSLGVQDTQAAVQEAVKRRQTHEESRRVYEKFKELGFTSINIDLIYGLPKQTRHTFAQTIQDILAMRPDRLALFSYAAVPWIKPHQKAIKATDLPSMEEKFAIYSQSRHSLIKAGYQAIGMDHFSLPEDPLTVAFHNKTLIRN
hemN_3    RVSLGVQDTQADVQEAVRRRQSHEESLKAYEKFKELAFQSINIDLIYGLPKQTKESFSKTIQDILAMYPDRLALFSFASVPWIKPHQKAMKASDMPSMEEKFAIYSQSRHLLTKAGYQAIGMDHFSLPHDPLTLAFKNKTLIRN
hemN_16   RVSLGVQDTQADVQEAVRRRQSHEESLKAYEKFKELGFQSINIDLIYGLPKQTKESFSKTIQDILAMYPDRLALFSFASVPWIKPHQKAMKASDMPSMEEKFAIYSQSRHLLTKAGYQAIGMDHFSLPHDPLTLAFKNKTLIRN
hemN_7    RVSLGIQDTQWEVQEAVRRRQSYEESLLAYKQFRDLNFTGINIDLIYGLPKQTQSSFKQTIQHILDMRPDRLALFSFAHVPWVKPHQKALRTKDLPSMEEKFAIYSQSRQALIQEGYQAIGLDHFSLPDDPLTIALKNKTLIRN
hemN_15   RVSLGIQDTQWEVQEAVRRRQSYEESLLAYQLFRDLKFTGINIDLIYGLPKQTQSSFKQTIEHILDMRPDRLALFSFAHVPWTKPHQKALRTKDLPSMEEKFAIYSQSRHTLIQEGYQAIGLDHFSLPDDPLTIALKNKTLIRN
hemN_19   RVSLGIQDTQWEVQEAVRRRQSYEESLLAYQLFRDLKFTGINIDLIYGLPKQTQSSFKQTIEHILDMRPDRLALFSFAHVPWAKPHQKALRTKDLPSMEEKFAIYSQSRHTLIQEGYQAIGLDHFSLPDDPLTIALKNKTLIRN
hemN_1    RVSLGIQDTQWEVQEAVRRRQSYEESLLAYQLFRDLKFTGINIDLIYGLPKQTQSSFKQTIEHILDMRPDRLALFSFAHVPWAKPHQKALRTKELPSMEEKFAIYSQSRHTLIQEGYQAIGLDHFSLPDDPLTIALKNKTLIRN
hemN_2    RVSLGIQDTQWEVQEAVRRRQSYEESLLAYQLFRDLKFTGINIDLIYGLPKQTQSSFKQTIEHILDMRPDRLALFSFAHVPWAKPHQKALRTKDLPSMEEKFAIYSQSRHTLIQEGYQAIGLDHFSLPDDPLTIALKNKTLIRN
hemN_18   RVSLGIQDTQWEVQEAVRRRQSYEESLLAYQLFRDLKFTGINIDLIYGLPKQTQSSFKQTIEHILDMRPDRLALFSFAHVPWAKPHQKALRTKDLPSMEEKFAIYSQSRHTLIQEGYQAIGLDHFSLPDDPLTIALKNKTLIHN
```

B. Nucleotide substitutions among three *C. abortus gatA* alleles (in boldface; *gatA*_5, *gatA*_18, *gatA*_13 ruminant and avian *C. abortus* alleles) (a)

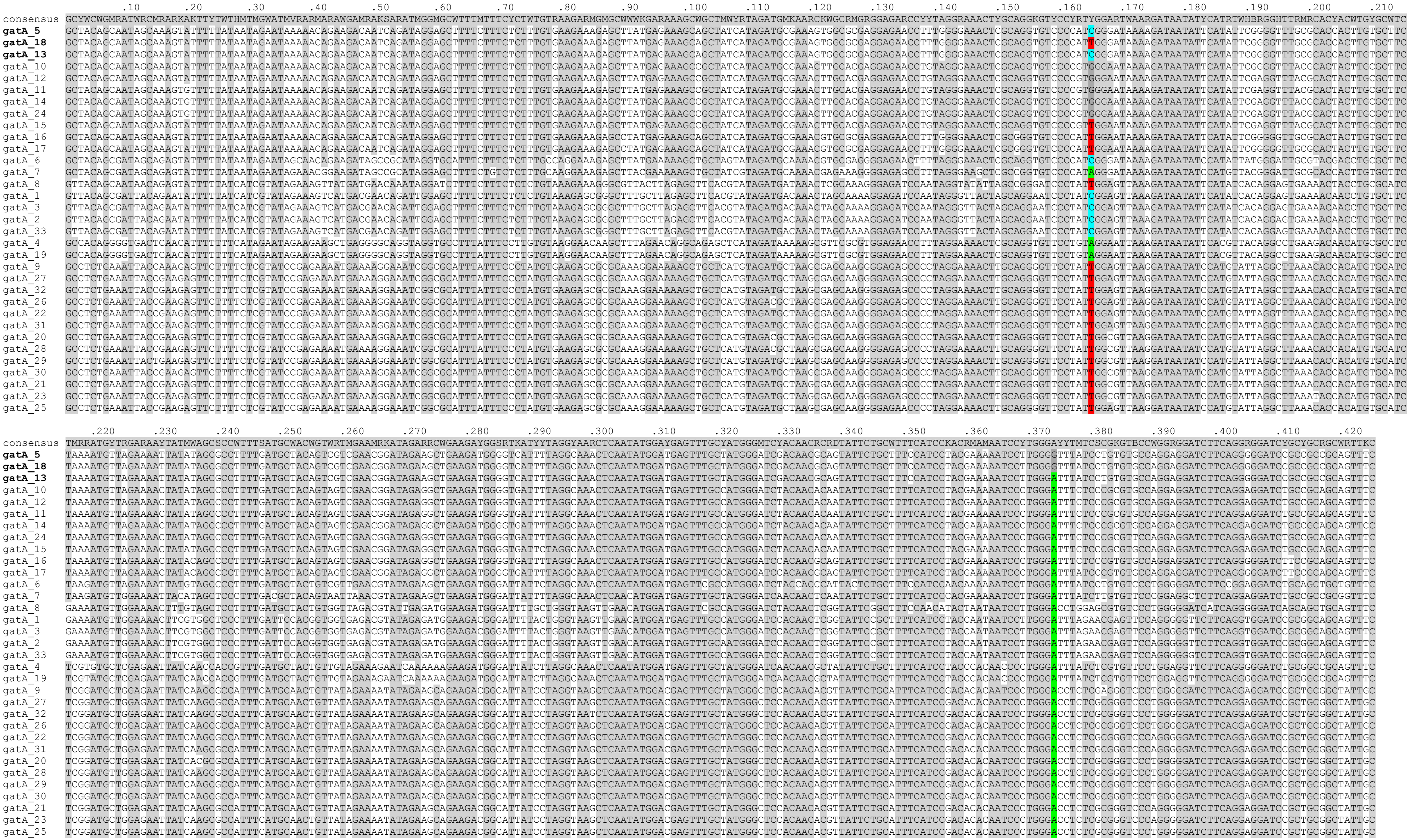

B. Amino-acid substitutions among three *C. abortus gatA*-encoded protein alleles (b)

```
                                                                                                           111111111111111111111111111111111111111111
                 11111111112222222222333333333344444444445555555555666666666677777777778888888888999999999900000000001111111111222222222233333333334444
        123456789012345678901234567890123456789012345678901234567890123456789012345678901234567890123456789012345678901234567890123456789012345678901
gatA_5  ATAIAKYFYNRIKTEDNQIGAFLSLCEERAYEKAAIIDAKVARGEPLGKLAGVPIGIKDNIHIRGLRTTCASKMLENYIAPFDATVVERIEAEDGVILGKLNMDEFAMGSTTQYSAFHPTKNPWGLSCVPGGSSGGSAAAV
gatA_18 ATAIAKYFYNRIKTEDNQIGAFLSLCEERAYEKAAIIDAKVARGEPLGKLAGVPIGIKDNIHIRGLRTTCASKMLENYIAPFDATVVERIEAEDGVILGKLNMDEFAMGSTTQYSAFHPTKNPWGLSCVPGGSSGGSAAAV
gatA_13 ATAIAKYFYNRIKTEDNQIGAFLSLCEERAYEKAAIIDAKVARGEPLGKLAGVPIGIKDNIHIRGLRTTCASKMLENYIAPFDATVVERIEAEDGVILGKLNMDEFAMGSTTQYSAFHPTKNPWDLSCVPGGSSGGSAAAV
gatA_10 ATAIAKYFYNRIKTEDNQIGAFLSLCEERAYEKAAIIDAKLARGEPVGKLAGVPVGIKDNIHIRGLRTTCASKMLENYIAPFDATVVERIEAEDGVILGKLNMDEFAMGSTTQYSAFHPTKNPWDFSRVPGGSSGGSAAAV
gatA_12 ATAIAKYFYNRIKTEDNQIGAFLSLCEERAYEKAAIIDAKLARGEPVGKLAGVPVGIKDNIHIRGLRTTCASKMLENYIAPFDATVVERIEAEDGVILGKLNMDEFAMGSTTQYSAFHPTKNPWDFSRVPGGSSGGSAAAV
gatA_11 ATAIAKCFYNRIKTEDNQIGAFLSLCEERAYEKAAIIDAKLARGEPVGKLAGVPVGIKDNIHIRGLRTTCASKMLENYIAPFDATVVERIEAEDGVILGKLNMDEFAMGSTTQYSAFHPTKNPWDFSRVPGGSSGGSAAAV
gatA_14 ATAIAKCFYNRIKTEDNQIGAFLSLCEERAYEKAAIIDAKLARGEPVGKLAGVPVGIKDNIHIRGLRTTCASKMLENYIAPFDATVVERIEAEDGVILGKLNMDEFAMGSTTQYSAFHPTKNPWDFSRVPGGSSGGSAAAV
gatA_24 ATAIAKCFYNRIKTEDNQIGAFLSLCEERAYEKAAIIDAKLARGEPVGKLAGVPVGIKDNIHIRGLRTTCASKMLENYIAPFDATVVERIEAEDGVILGKLNMDEFAMGSTTQYSAFHPTKNPWDFSRVPGGSSGGSAAAV
gatA_15 ATAIAKYFYNRIKTEDNQIGAFLSLCEERAYEKAAIIDAKLARGEPVGKLAGVPVGIKDNIHIRGLRTTCASKMLENYIAPFDATVVERIEAEDGVILGKLNMDEFAMGSTTQYSAFHPTKNPWDFSRVPGGSSGGSAAAV
gatA_16 ATAIAKYFYNRIKTEDNQIGAFLSLCEERAYEKAAIIDAKRARGEPLGKLAGVPIGIKDNIHIRGLRTTCASKMLENYTAPFDATVVERIEAEDGVILGKLNMDEFAMGSTTQYSAFHPTKNPWDFSRVPGGSSGGSSAAV
gatA_17 ATAIAKYFYNRIKTEDNQIGAFLSLCEERAYEKAAIIDAKRARGEPLGKLAGVPIGIKDNIHIRGLRTTCASKMLENYTAPFDATVVERIEAEDGVILGKLNMDEFAMGSTTQYSAFHPTKNPWDLSRVPGGSSGGSSAAV
gatA_6  ATAIAEYFYNRIATEDSRIGAFLSLCQERAYEKAASIDAKRARGELLGKLAGVPIGIKDNIHIMGLRTTCASKMLENYVAPFDATVVERIEAEDGIILGKLNMDEFAMGSTTHYSAFHPTKNPWDLSCVPGGSSGGSAAAV
gatA_7  ATAIAEYFYNRIETEDSRIGAFLSLCKERAYEKAAIVDAKRERGEPLGKLAGVPIGIKDNIHVTGLRTTCASKMLENYIAPFDATVIKRIEAEDGIILGKLNMDEFAMGSTTQYSAFHPTKNPWDLSCVPGGSSGGSAAAV
gatA_8  VTAITEYFYHRIESYDEQIGSFLSLCKERALLRASRIDDKLAKGDPIGILAGIPIGVKDNIHITGVKTTCASKMLENFVAPFDATVVRRIEMEDGILLGKLNMDEFAMGSTTRYSAFQHTNNPWDLERVPGGSSGGSAAAV
gatA_1  VTAITEYFYHRIESHDEQIGAFLSLCKERALLRASRIDDKLAKGDPIGLLAGIPIGVKDNIHITGVKTTCASKMLENFVAPFDSTVVRRIEMEDGILLGKLNMDEFAMGSTTRYSAFHPTNNPWDLERVPGGSSGGSAAAV
gatA_3  VTAITEYFYHRIESHDEQIGAFLSLCKERALLRASRIDDKLAKGDPIGLLAGIPIGVKDNIHITGVKTTCASKMLENFVAPFDSTVVRRIEMEDGILLGKLNMDEFAMGSTTRYSAFHPTNNPWDLERVPGGSSGGSAAAV
gatA_2  VTAITEYFYHRIESHDEQIGAFLSLCKERALLRASRIDDKLAKGDPIGLLAGIPIGVKDNIHITGVKTTCASKMLENFVAPFDSTVVRRIEMEDGILLGKLNMDEFAMGSTTRYSAFHPTNNPWDLERVPGGSSGGSAAAV
gatA_33 VTAITEYFYHRIESHDEQIGAFLSLCKERALLRASRIDDKLAKGDPIGLLAGIPIGVKDNIHITGVKTTCASKMLENFVAPFDSTVVRRIEMEDGILLGKLNMDEFAMGSTTRYSAFHPTNNPWDLERVPGGSSGGSAAAV
gatA_4  ATGVTQHFFHRIEEAEGQVGAFISLCKEQALEQAELIDKKRSRGEPLGKLAGVPVGIKDNIHVTGLKTTCASRVLENYQPPFDATVVERIKKEDGIILGKLNMDEFAMGSTTLYSAFHPTHNPWDLSRVPGGSSGGSAAAV
gatA_19 ATGVTQHFFHRIEEAEGQVGAFISLCKEQALEQAELIDKKRSRGEPLGKLAGVPVGIKDNIHVTGLKTTCASRMLENYQPPFDATVVERIKKEDGIILGKLNMDEFAMGSTTLYSAFHPTHNPWDLSRVPGGSSGGSAAAV
gatA_9  ASEITKEFFSRIRENEKEIGAFISLCEERAKEKAAHVDAKRARGEPLGKLAGVPIGVKDNIHVLGLNTTCASRMLENYQAPFHATVIENIEAEDGIILGKLNMDEFAMGSTTRYSAFHPTHNPWDLSRVPGGSSGGSAAAI
gatA_27 ASEITEEFFSRIRENEKEIGAFISLCEERAKEKAAHVDAKRARGEPLGKLAGVPIGVKDNIHVLGLNTTCASRMLENYQAPFHATVIENIEAEDGIILGKLNMDEFAMGSTTRYSAFHPTHNPWDLSRVPGGSSGGSAAAI
gatA_32 ASEITEEFFSRIRENEKEIGAFISLCEERAKEKAAHVDAKRARGEPLGKLAGVPIGVKDNIHVLGLNTTCASRMLENYQAPFHATVIENIEAEDGIILGNLNMDEFAMGSTTRYSAFHPTHNPWDLSRVPGGSSGGSAAAI
gatA_26 ASEITEEFFSRIRENEKEIGAFISLCEERAKEKAAHVDAKRARGEPLGKLAGVPIGVKDNIHVLGLNTTCASRMLENYQAPFHATVIENIEAEDGIILGKLNMDEFAMGSTTRYSAFHPTHNPWDLSRVPGGSSGGSAAAI
gatA_22 ASEITEEFFSRIRENEKEIGAFISLCEERAKEKAAHVDAKRARGEPLGKLAGVPIGVKDNIHVLGLNTTCASRMLENYQAPFHATVIENIEAEDGIILGKLNMDEFAMGSTTRYSAFHPTHNPWDLSRVPGGSSGGSAAAI
gatA_31 ASEITEEFFSRIRENEKEIGAFISLCEERAKEKAAHVDAKRARGEPLGKLAGVPIGVKDNIHVLGLNTTCASRMLENYQAPFHATVIENIEAEDGIILGKLNMDEFAMGSTTRYSAFHPTHNPWDLSRVPGGSSGGSAAAI
gatA_20 ASEITEEFFSRIRENEKEIGAFISLCEERAKEKAAHVDAKRARGEPLGKLAGVPIGVKDNIHVLGLNTTCASRMLENYHAPFHATVIENIEAEDGIILGKLNMDEFAMGSTTRYSAFHPTHNPWDLSRVPGGSSGGSAAAI
gatA_28 ASEITEEFFSRIRENEKEIGAFISLCEERAKEKAAHVDAKRARGEPLGKLAGVPIGVKDNIHVLGLNTTCASRMLENYQAPFHATVIENIEAEDGIILGKLNMDEFAMGSTTRYSAFHPTHNPWDLSRVPGGSSGGSAAAI
gatA_29 ASEITEEFFSRIRENEKEIGAFISLCEERAKEKAAHVDAKRARGEPLGKLAGVPIGVKDNIHVLGLNTTCASRMLENYQAPFHATVIENIEAEDGIILGKLNMDEFAMGSTTRYSAFHPTHNPWDLSRVPGGSSGGSAAAI
gatA_30 ASEITEEFFSRIRENEKEIGAFISLCEERAKEKAAHVDAKRARGEPLGKLAGVPIGVKDNIHVLGLNTTCASRMLENYQAPFHATVIENIEAEDGIILGKLNMDEFAMGSTTRYSAFHPTHNPWDLSRVPGGSSGGSAAAI
gatA_21 ASEITEEFFSRIRENEKEIGAFISLCEERAKEKAAHVDAKRARGEPLGKLAGVPIGVKDNIHVLGLNTTCASRMLENYQAPFHATVIENIEAEDGIILGKLNMDEFAMGSTTRYSAFHPTHNPWDLSRVPGGSSGGSAAAI
gatA_23 ASEITEEFFSRIRENEKEIGAFISLCEERAKEKAAHVDAKRARGEPLGKLAGVPIGVKDNIHVLGLNTTCASRMLENYQAPFHATVIENIEAEDGIILGKLNMDEFAMGSTTRYSAFHPTHNPWDLSRVPGGSSGGSAAAI
gatA_25 ASEITEEFFSRIRENEKEIGAFISLCEERAKEKAAHVDAKRARGEPLGKLAGVPIGVKDNIHVLGLNTTCASRMLENYQAPFHATVIENIEAEDGIILGKLNMDEFAMGSTTRYSAFHPTHNPWDLSRVPGGSSGGSAAAI
```

C. Nucleotide substitutions among four *C. abortus fumC* alleles (in boldface; *fumC*_5, *fumC*_18, *fumC*_17, *fumC*_14 ruminant or avian *C. abortus* alleles) (a)

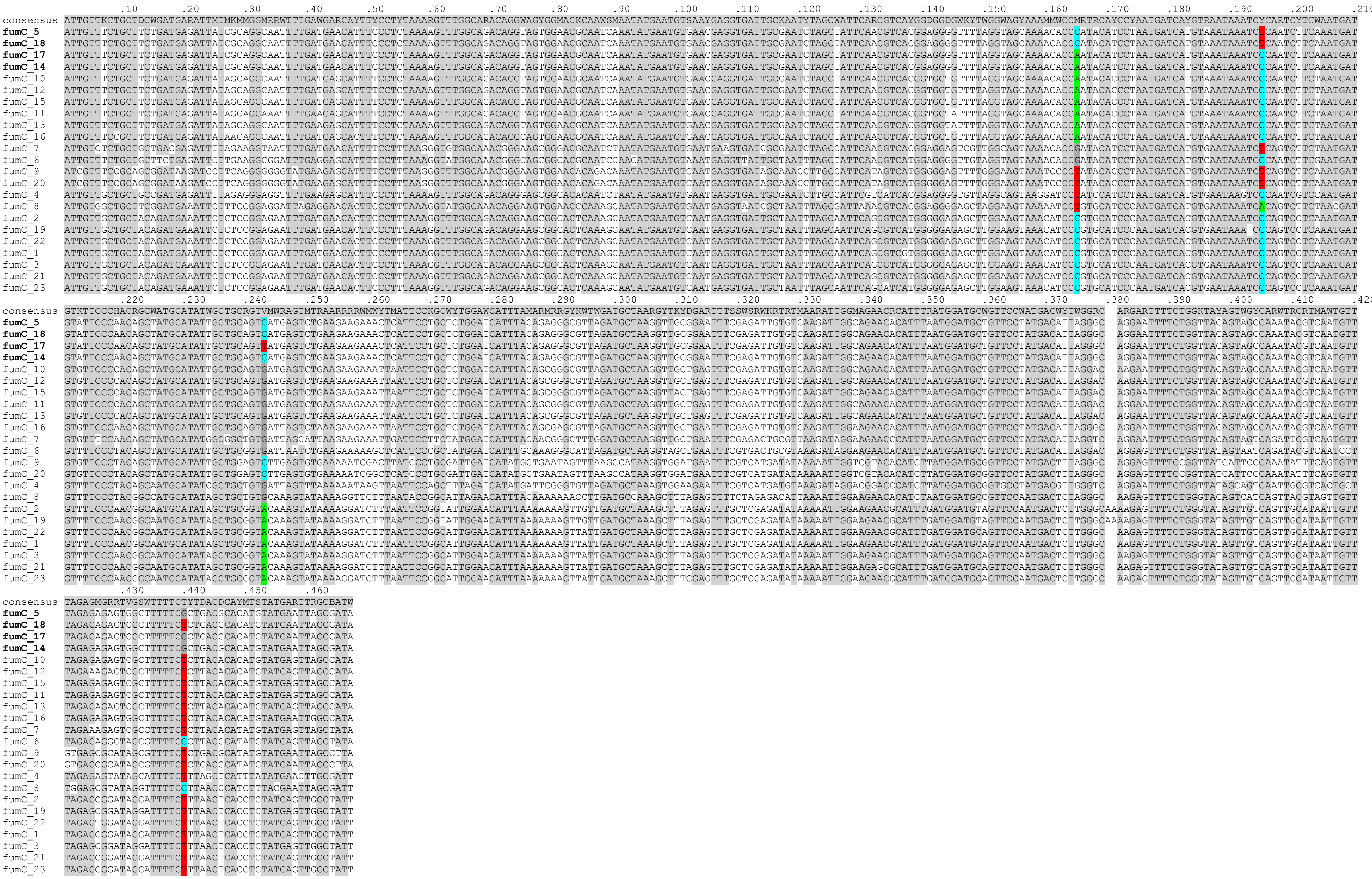

C. Sequence alignment among *C. abortus fumC*-encoded protein alleles (b)

```
                                                                                                           11111111111111111111111111111111111111111111111111111111
                 111111111122222222223333333333444444444455555555556666666666777777777788888888889999999999000000000011111111112222222222333333333344444444445555555
        12345678901234567890123456789012345678901234567890123456789012345678901234567890123456789012345678901234567890123456789012345678901234567890123456789012345
fumC_5  IVSASDEIIAGNFDEHFPLKVWQTGSGTQSNMNVNEVIANLAIQRHGGVLGSKTPIHPNDHVNKSQSSNDVFPTAMHIAAVMSLKKKLIPALDHLQRALDAKVAEFRDCVKIGRTHLMDAVPMTLGQEFSGYSSQIRQCLERVAFSLTHMYELAI
fumC_18 IVSASDEIIAGNFDEHFPLKVWQTGSGTQSNMNVNEVIANLAIQRHGGVLGSKTPIHPNDHVNKSQSSNDVFPTAMHIAAVMSLKKKLIPALDHLQRALDAKVAEFRDCVKIGRTHLMDAVPMTLGQEFSGYSSQIRQCLERVAFSLTHMYELAI
fumC_17 IVSASDEIIAGNFDEHFPLKVWQTGSGTQSNMNVNEVIANLAIQRHGGVLGSKTPIHPNDHVNKSQSSNDVFPTAMHIAAVMSLKKKLIPALDHLQRALDAKVAEFRDCVKIGRTHLMDAVPMTLGQEFSGYSSQIRQCLERVAFSLTHMYELAI
fumC_14 IVSASDEIIAGNFDEHFPLKVWQTGSGTQSNMNVNEVIANLAIQRHGGVLGSKTPIHPNDHVNKSQSSNDVFPTAMHIAAVMSLKKKLIPALDHLQRALDAKVAEFRDCVKIGRTHLMDAVPMTLGQEFSGYSSQIRQCLERVAFSLTHMYELAI
fumC_10 IVSASDEIIAGNFDEHFPLKVWQTGSGTQSNMNVNEVIANLAIQRHGGVLGSKTPIHPNDHVNKSQSSNDVFPTAMHIAAVMSLKKKLIPALDHLQRALDAKVAEFRDCVKIGRTHLMDAVPMTLGQEFSGYSSQIRQCLERVAFSLTHMYELAI
fumC_12 IVSASDEIIAGNFDEHFPLKVWQTGSGTQSNMNVNEVIANLAIQRHGGVLGSKTPIHPNDHVNKSQSSNDVFPTAMHIAAVMSLKKKLIPALDHLQRALDAKVAEFRDCVKIGRTHLMDAVPMTLGQEFSGYSSQIRQCLERVAFSLTHMYELAI
fumC_15 IVSASDEIIAGNFDEHFPLKVWQTGSGTQSNMNVNEVIANLAIQRHGGVLGSKTPIHPNDHVNKSQSSNDVFPTAMHIAAVMSLKKKLIPALDHLQRALDAKVTEFRDCVKIGRTHLMDAVPMTLGQEFSGYSSQIRQCLERVAFSLTHMYELAI
fumC_11 IVSASDEIIAGNFEEHFPLKVWQTGSGTQSNMNVNEVIANLAIQRHGGILGSKTPIHPNDHVNKSQSSNDVFPTAMHIAAVMSLKKKLIPALDHLQRALDAKVAEFRDCVKIGRTHLMDAVPMTLGQEFSGYSSQIRQCLERVAFSLTHMYELAI
fumC_13 IVSASDEIIAGNFEEHFPLKVWQTGSGTQSNMNVNEVIANLAIQRHGGILGSKTPIHPNDHVNKSQSSNDVFPTAMHIAAVMSLKKKLIPALDHLQRALDAKVAEFRDCVKIGRTHLMDAVPMTLGQEFSGYSSQIRQCLERVAFSLTHMYELAI
fumC_16 IVSASDEIITGNFDEHFPLKVWQTGSGTQSNMNVNEVIANLAIQRHGGVLGSKTPIHPNDHVNKSQSSNDVFPTAMHIAAVISLKKKLIPALDHLQRALDAKVAEFRDCVKIGRTHLMDAVPMTLGQEFSGYSSQIRQCLERVAFSLTHMYELAI
fumC_7  IVSAADEILEGNFDEHFPLRVWQTGSGTQSNMNVNEVIANLAIQRHGGVVGSKTPIHPNDHVNKSQSSNDVFPTAMHMAAVISIKKKLIPSMDHLQRALDAKVAEFRDCVKIGRTHLMDAVPMTLGQEFSGYSSQIRQCLERVAFSLTHMYELAI
fumC_6  IVSAASEILEGGFEEHFPLKVWQTGSGTQSNMNVNEVIANLAIQRHGGVVGSKTPIHPNDHVNKSQSSNDVFPTAMHIAAVINLKKKLIPAMDHLQRALDAKVAEFRDCVKIGRTHLMDAVPMTLGQEFSGYSNQIRQSLERVAFSLTHMYELAI
fumC_9  IVSAADKILQGGYEEHFPLRVWQTGSGTQTNMNVNEVIANLAIHSHGGVLGSKSPIHPNDHVNKSQSSNDVFPTAMHIAGVLSVKNRLIPAIDHMLNSLSHKVDEFRHDIKIGRTHLMDAVPMTLGQEFSGYHSQIFQCCERIAFSLTHMYELAL
fumC_20 IVSAADKILQGGYEEHFPLRVWQTGSGTQTNMNVNEVIANLAIHSHGGVLGSKSPIHPNDHVNKSQSSNDVFPTAMHIAGVLSVKNRLIPAIDHMLNSLSHKVDEFRHDIKIGRTHLMDAVPMTLGQEFSGYHSQIFQCCERIAFSLTHMYELAL
fumC_4  IVAAADEILEGGFEEHFPLKVWQTGSGTQSNMNVNEVIANLAIRHHGGVLGSKDPIHPNDHVNKSQSSNDVFPTAMHIAAVISLKNKLIPALDHMIRVLDAKVEEFRHDVKIGRTHLMDAVPMTLGQEFSGYSSQLRHCLESIAFSLAHLYELAI
fumC_8  IVAASDEILSGGLEEHFPLKVWQTGSGTQSNMNVNEVIANLAIKRHGGELGSKNPVHPNDHVNKSQSSNDVFPTAMHIAAVQSIKGSLIPALEHLQKNLDAKALEFSRDIKIGRTHLMDAVPMTLGQEFSGYSHQLRSCLERIGFSLTHLYELAI
fumC_1  IVAATDEILSGEFDEHFPLKVWQTGSGTQSNMNVNEVIANLAIQRRGGELGSKHPVHPNDHVNKSQSSNDVFPTAMHIAAVQSIKGSLIPALEHLKKVIDAKALEFARDIKIGRTHLMDAVPMTLGQEFSGYSCQLHNCLERIGFSLTHLYELAI
fumC_21 IVAATDEILSGEFDEHFPLKVWQTGSGTQSNMNVNEVIANLAIQRHGGELGSKHPVHPNDHVNKSQSSNDVFPTAMHIAAVQSIKGSLIPALEHLKKVIDAKALEFARDIKIGRAHLMDAVPMTLGQEFSGYSCQLHNCLERIGFSLTHLYELAI
fumC_3  IVAATDEILSGEFDEHFPLKVWQTGSGTQSNMNVNEVIANLAIQRHGGELGSKHPVHPNDHVNKSQSSNDVFPTAMHIAAVQSIKGSLIPALEHLKKVIDAKALEFARDIKIGRTHLMDAVPMTLGQEFSGYSCQLHNCLERIGFSLTHLYELAI
fumC_22 IVAATDEILSGEFDEHFPLKVWQTGSGTQSNMNVNEVIANLAIQRHGGELGSKHPVHPNDHVNKSQSSNDVFPTAMHIAAVQSIKGSLIPALEHLKKVIDAKALEFARDIKIGRTHLMDAVPMTLGQEFSGYSCQLHNCLEWIGFSLTHLYELAI
fumC_23 IVAATDEILSGEFDEHFPLKVWQTGSGTQSNMNVNEVIANLAIQHHGGELGSKHPVHPNDHVNKSQSSNDVFPTAMHIAAVQSIKGSLIPALEHLKKVIDAKALEFARDIKIGRTHLMDAVPMTLGQEFSGYSCQLHNCLERIGFSLTHLYELAI
```

D. Nucleotide substitutions among three *C. abortus enoA* alleles (in boldface; *enoA*_8, *enoA*_16, *enoA*_14 ruminant or avian *C. abortus* alleles) (a)

```
                .10       .20       .30       .40       .50       .60       .70       .80       .90      .100      .110      .120      .130      .140      .150      .160      .170      .180      .190      .200      .210
consensus GGBATGCAYGCHRMHAAYGGYYTSSAATTYCAAGAATTTATGATYCGYCCDATWRGVGCDCMYTCYMTRMMMGARGCKGTRYSBATGGGDGCWGAWGTHTTYMAYRCYTTRAAAAMMTCYTMMAKDMWMRWCABYTVKCTACAGGWGTDGGHGATGAAGGMGGMTTYGCTCCDMAHYTWVVHTCYAAYKCHGAAGCTYTRGABCTWCTY
enoA_8    GGTATGCATGCAAACAACGGCCTGCAATTTCAAGAATTTATGATTCGCCCTATAGGCGCACATTCTCTACAAGAAGCTGTACGTATGGGTGCCGATGTGTTCCATACATTGAAAAACCTTCTCAATGATAAACATCTCGCTACAGGAGTGGGAGATGAAGGCGGATTTGCTCCACAATTAAAATCTAACTCTGAAGCTTTAGACCTTCTT
enoA_16   GGTATGCATGCAAACAACGGCCTGCAATTTCAAGAATTTATGATTCGCCCTATAGGCGCACATTCTCTACAAGAAGCTGTACGTATGGGTGCCGATGTGTTCCATACATTGAAAAACCTTCTCAATGATAAACATCTCGCTACAGGAGTGGGAGATGAAGGCGGATTTGCTCCACAATTAAAATCTAACTCTGAAGCTTTAGACCTTCTT
enoA_14   GGTATGCATGCAAACAACGGCCTGCAATTTCAAGAATTTATGATTCGCCCTATAGGCGCACATTCTCTACAAGAAGCTGTACGTATGGGTGCCGATGTGTTCCATACATTGAAAAACCTTCTCAATGATAAACATCTCGCTACAGGAGTGGGAGATGAAGGCGGATTTGCTCCACAATTAAAATCTAACTCTGAAGCTTTAGACCTTCTT
enoA_13   GGCATGCATGCAGACAATGGTCTTCAATTTCAAGAATTTATGATTCGCCCTATAGGTGCACATTCTCTAAAAGAAGCTGTGCGTATGGGTGCTGATGTTTTCCATGCGTTGAAAAAACTGCTCAACGATAGACATCTCGCTACAGGAGTTGGAGATGAAGGCGGATTTGCTCCAAACTTAACATCTAACTCTGAAGCTTTAGACCTTCTT
enoA_15   GGCATGCATGCAGACAATGGTCTTCAATTTCAAGAATTTATGATTCGCCCTATAGGTGCACATTCTCTAAAAGAAGCTGTGCGTATGGGTGCTGATGTTTTCCATGCGTTGAAAAAACTGCTCAACGATAGACATCTCGCTACAGGAGTTGGAGATGAAGGCGGATTTGCTCCAAACTTAACATCTAACTCTGAAGCTTTAGACCTTCTT
enoA_10   GGTATGCATGCCAATAATGGACTACAATTCCAAGAATTTATGATTCGTCCTATCGGAGCAACATCAATAAAAGAGGCGGTACGTATGGGAGCAGATGTTTTTCACACTCTGAAAAAACTCCTCAACGATAAGAACCTCGCTACGGGAGTGGGAGATGAGGGAGGATTTGCTCCTCAACTCAAATCCAACTCTGAAGCTTTAGATCTTCTT
enoA_9    GGGATGCACGCAAATAACGGTTTGCAATTCCAAGAGTTTATGATTCGTCCTATCGGAGCAACTTCTCTAAAAGAAGCTGTACGGATGGGCGCTGATGTTTTTCATTCTTTGAAAAATATCCTCAATGAAAAAAACTTAGCTACAGGGGTTGGTGATGAAGGAGGCTTTGCTCCACAACTAAAATCTAACTCTGAAGCATTAGATCTTCTT
enoA_5    GGCATGCATGCCGATAACGGCTTGGGGTTCCAAGAATTTATGATCCGTCCTATTGGAGCCTCTTCCATCAAAGAAGCTGTCAACATGGGTGCTGACGTTTTTCATACTTTGAAAAAATTACTCCATGAAAGAGGCTTATCTACTGGAGTGGGTGACGAAGGAGGCTTCGCCCCGAATCTTGCTTCTAATGAAGAAGCTCTAGAGCTCCTA
enoA_6    GGCATGCATGCCGATAACGGCTTGGAGTTCCAAGAATTTATGATCCGTCCTATTGGAGCCTCTTCCATCAAAGAAGCTGTCAACATGGGTGCTGACGTTTTTCATACTTTGAAAAAATTACTCCATGAAAGAGGCTTATCTACTGGAGTGGGTGACGAAGGAGGCTTCGCCCCGAATCTTGCTTCTAATGAAGAAGCTCTAGAGCTCCTA
enoA_7    GGCATGCATGCCGATAACGGCTTGGAGTTCCAAGAATTTATGATCCGTCCTATTGGAGCCTCTTCCATCAAAGAAGCTGTCAACATGGGTGCTGACGTTTTTCATACTTTGAAAAAATTACTCCATGAAAGAGGCTTATCTACTGGAGTGGGTGACGAAGGAGGCTTCGCCCCGAATCTTGCTTCTAATGAAGAAGCTCTAGAGCTCCTA
enoA_17   GGCATGCATGCCGATAACGGCTTGGAGTTCCAAGAATTTATGATCCGTCCTATTGGAGCCTCTTCCATCAAAGAAGCTGTCAACATGGGTGCTGACGTTTTTCATACTTTGAAAAAATTACTCCATGAAAGAGGCTTATCTACTGGAGTGGGTGACGAAGGAGGCTTCGCCCCGAATCTTGCTTCTAATGAAGAAGCTCTAGAGCTCCTA
enoA_12   GGGATGCACGCAGATAATGGCCTGGAATTTCAAGAATTTATGATCCGTCCGATTGGGGCGCACTCTATGCACGAGGCGGTATCTATGGGGGCGGATGTATTCCATGCTTTGAAAAACCTCTTAAAGTCACGTCATTTAGCTACAGGAGTAGGCGATGAAGGAGGCTTTGCTCCGAACTTACGCTCCAATGCAGAAGCTTTAGAGCTTCTC
enoA_18   GGGATGCACGCAGATAATGGCCTGGAATTTCAAGAATTTATGATCCGTCCGATTGGGGCGCACTCTATGCACGAGGCGGTATCTATGGGGGCGGATGTATTCCATGCTTTGAAAAACCTCTTAAAGTCACGTCATTTAGCTACAGGAGTAGGCGATGAAGGAGGCTTTGCTCCGAACTTACGCTCCAATGCAGAAGCTTTAGAGCTTCTC
enoA_22   GGGATGCACGCAGATAATGGCCTGGAATTTCAAGAATTTATGATCCGTCCGATTGGGGCGCACTCTATGCACGAGGCGGTATCTATGGGGGCGGATGTATTCCATGCTTTGAAAAACCTCTTAAAGTCACGTCATTTAGCTACAGGAGTAGGCGATGAAGGAGGCTTTGCTCCGAACTTACGCTCCAATGCAGAAGCTTTAGAGCTTCTC
enoA_23   GGGATGCACGCAGATAATGGCCTGGAATTTCAAGAATTTATGATCCGTCCGATTGGGGCGCACTCTATGCACGAGGCGGTATCTATGGGGGCAGATGTATTCCATGCTTTGAAAAACCTCTTAAAGTCACGTCATTTAGCTACAGGAGTAGGCGATGAAGGAGGCTTTGCTCCGAACTTACGCTCCAATGCAGAAGCTTTAGAGCTTCTC
enoA_19   GGGATGCACGCAGATAATGGCCTGGAATTTCAAGAATTTATGATCCGTCCGATTGGGGCGCACTCTATGCACGAGGCGGTATCTATGGGGGCAGATGTATTCCATGCCTTGAAAAACCTCTTAAAGTCACGTCATTTAGCTACAGGAGTAGGCGATGAAGGAGGCTTTGCTCCGAACTTACGCTCCAATGCAGAAGCTTTAGAGCTTCTC
enoA_20   GGGATGCACGCAGATAATGGCCTGGAATTTCAAGAATTTATGATCCGTCCGATTGGAGCGCACTCTATGCACGAGGCGGTATCTATGGGGGCAGATGTATTCCATGCCTTGAAAAACCTCTTAAAGTCACGTCATTTAGCTACAGGAGTAGGCGATGAAGGAGGCTTTGCTCCGAACTTACGCTCCAATGCAGAAGCTTTAGAGCTTCTC
enoA_21   GGGATGCACGCAGATAATGGCCTGGAATTTCAAGAATTTATGATCCGTCCGATTGGAGCGCACTCTATGCACGAGGCGGTATCTATGGGGGCAGATGTATTCCATGCCTTGAAAAACCTCTTAAAGTCACGTCATTTAGCTACAGGAGTAGGCGATGAAGGAGGCTTTGCTCCGAACGTACGCTCCAATGCAGAAGCTTTAGAGCTTCTC
enoA_11   GGCATGCATGCAACAAATGGCCTCCAATTTCAAGAATTTATGATCCGTCCTATTAGCGCTCCTTCCCTAACAGAAGCTGTGCGCATGGGAGCAGAAGTTTTTCACACTTTGAAAAAAATCTTACAAAATCGGCAACTTTCTACAGGAGTCGGAGATGAAGGCGGATTCGCTCCCCAACTCGCTTCCAATTCAGAGGCGCTTGACCTTCTC
enoA_2    GGTATGCATGCTACAAATGGTCTCCAATTCCAAGAATTTATGATTCGTCCAATTAGCGCTCCTTCTCTAAAAGAGGCTGTGCGGATGGGAGCAGAAGTCTTCAACGCCTTAAAAAAAATCTTACAGAATCGACAGCTGGCTACAGGTGTTGGTGATGAAGGCGGATTTGCTCCTAATCTTGCCTCTAATGCCGAAGCTCTGGATCTACTC
enoA_3    GGTATGCATGCTACAAATGGTCTCCAATTCCAAGAATTTATGATTCGTCCAATTAGCGCTCCTTCTCTAACAGAGGCTGTGCGGATGGGAGCAGAAGTCTTCAACGCCTTAAAAAAAATCTTACAGAATCGACAGCTGGCTACAGGTGTTGGTGATGAAGGCGGATTTGCTCCTAATCTTGCCTCTAATGCCGAAGCTCTGGATCTACTC
enoA_25   GGTATGCATGCTACAAATGGTCTCCAATTCCAAGAATTTATGATTCGTCCAATTAGCGCTCCTTCTCTAACAGAGGCTGTGCGGATGGGAGCAGAAGTCTTCAACGCCTTAAAAAAAATCTTACAGAATCGACAGCTGGCTACAGGTGTTGGTGATGAAGGCGGATTTGCTCCTAATCTTGCCTCTAATGCCGAAGCTCTGGATCTACTC
enoA_4    GGTATGCATGCTACAAATGGTCTCCAATTCCAAGAATTTATGATTCGTCCAATTAGCGCTCCTTCTCTAACAGAGGCTGTGCGGATGGGAGCAGAAGTCTTCAACGCCTTAAAAAAAATCTTACAAAATCGACAGCTGGCTACAGGTGTTGGTGATGAAGGCGGATTTGCTCCTAATCTTGCCTCTAATGCCGAAGCTCTGGATCTACTC
enoA_1    GGTATGCATGCTACAAATGGTCTCCAATTCCAAGAATTTATGATTCGTCCAATTAGCGCTCCTTCTCTAACAGAGGCTGTGCGGATGGGAGCAGAAGTCTTCAACGCCTTAAAAAAAATCTTACAGAATCGACAGCTGGCTACAGGTGTTGGTGATGAAGGTGGATTTGCTCCTAATCTTGCCTCTAATGCCGAAGCTCTGGATCTACTC
enoA_24   GGTATGCATGCTACAAATGGTCTCCAATTCCAAGAATTTATGATTCGTCCAATTAGCGCTCCTTCTCTAACAGAGGCTGTGCGGATGGGAGCAGAAGTCTTCAACGCCTTAAAAAAAATCTTACAGAATCGACAGCTGGCTACAGGTGTCGGTGATGAAGGCGGATTTGCTCCTAATCTTGCCTCTAATGCCGAAGCTCTGGATCTACTC

               .220      .230      .240      .250      .260      .270      .280      .290      .300      .310      .320      .330      .340      .350      .360      .370      .380
consensus DTRMYWGCWATHGAARMHGCAGGHTTYVHVCCTRGVRARGADRTYTCTYTAGCTCTHGAYTGYGCWGCWTCYTCTTTYTAYRAWRCMVMRRMWRAAACYTAYSAHGGSARRHMBTMTSMWGMWCARRTVGVBATWCTYRMMGADYTHTGYGAKCRBTWTCCTATAGAYTCY
enoA_8    GTATTAGCTATTGAGAAAGCCGGTTTCCAGCCTGGCGAGGAGATCTCTTTAGCTCTTGACTGTGCTGCATCTTCTTTCTACGATACAAAAACAGAAACTTACGCAGGGAAAAACTCTCAAGAACAAGTCGGCATACTCGCCGATCTTTGTGATCGTTATCCTATAGACTCT
enoA_16   GTATTAGCTATTGAGAAAGCCGGTTTCCAGCCTGGCGAGGAGATCTCTTTAGCTCTTGACTGTGCTGCATCTTCTTTCTATGATACAAAAACAGAAACTTACGCAGAGAAAAACTCTCAAGAACAAGTCGGCATACTCGCCGATCTTTGTGATCGTTATCCTATAGACTCT
enoA_14   GTATTAGCTATTGAGAAAGCCGGTTTCCAGCCTGGCGAAGAGATCTCTTTAGCTCTTGACTGTGCTGCATCTTCTTTCTACGATACAAAAACAGAAACTTACGCAGGGAAAAACTCTCAAGAACAAGTCGGCATACTCGCCGATCTTTGTGATCGTTATCCTATAGACTCT
enoA_13   TTACTAGCTATTGAAAAAGCAGGTTTCCAGCCTGGCGAGGAGATCTCTTTAGCTCTTGACTGTGCTGCATCTTCTTTCTACGATACAAAAACAAAAACTTACGAAGGGAAAAACTCTCAAGAACAAGTCAGCATACTCGCCGATCTTTGTGATCGTTATCCTATTGACTCT
enoA_15   TTACTAGCTATTGAAAAAGCAGGTTTCCAGCCTGGCGAGGAGATCTCTTTAGCTCTTGACTGTGCTGCATCTTCTTTCTACGATACAAAAACAAAAACTTACGAAGGGAAAAACTCTCAAGAACAAGTCAGCATACTCGCCGATCTTTGTGACCGTTATCCTATTGACTCT
enoA_10   GTATTAGCTATTGAAAAATCTGGTTTCCAACCGGGGAAAGAAATTTCACTAGCTCTTGATTGCGCAGCATCTTCTTTCTATGATACCAAAACAAAAACTTACGAGGGTAAGAGCTATCAAGAACAAGTAGAAATACTTGCAGATCTTTGCGACCGTTATCCTATTGATTCC
enoA_9    GTACTTGCTATCGAAAAAGCAGGTTTCCAACCTGGAGAAGACATCTCCCTAGCTCTCGATTGCGCAGCTTCTTCCTTCTATGATACGAAAGCAGAAACCTATGATGGAAAGAGCTATGAAGAACAAGTGAACATACTTGCCGATCTCTGTGATCGCTATCCTATCGATTCT
enoA_5    TTGCTGGCTATTGAAAAAGCAGGCTTTACTCCAGGAAAAGATATATCGCTAGCCTTAGACTGCGCAGCATCCTCATTCTATAACGTAAAAACAGGCACGTATGATGGGAGGCACTATGAAGAGCAAATCGCAATCCTTTCTAATTTATGTGATCGCTATCCTATAGACTCC
enoA_6    TTGCTGGCTATTGAAAAAGCAGGCTTTACTCCAGGAAAAGATATATCGCTAGCCTTAGACTGCGCAGCATCCTCATTCTATAACGTAAAAACAGGCACGTATGATGGGAGGCACTATGAAGAGCAAATCGCAATCCTTTCTAATTTATGTGATCGCTATCCTATAGACTCC
enoA_7    TTGCTGGCTATTGAAAAAGCAGGCTTTACTCCAGGAAAAGATATATCGCTAGCCTTAGACTGCGCAGCGTCCTCATTCTATAACGTAAAAACAGGCACGTATGATGGGAGGCACTATGAAGAGCAAATCGCAATCCTTTCTAATTTATGTGATCGCTATCCTATAGACTCC
enoA_17   TTGCTGGCTATTGAAAAAGCAGGCTTTACTCCAGGAAAAGATATATCGCTAGCCTTAGACTGCGCAGCATCCTCATTCTATAACGTAAAAACAGGCACGTATGATGGAAGGCACTATGAAGAGCAAATCGCTATCCTTTCTAATTTATGTGATCGCTATCCTATAGACTCC
enoA_12   ATGACTGCAATAGAAGCCGCAGGTTTTGTCCCTGGGGAGGAAGTTTCTTTAGCTCTAGATTGCGCAGCATCTTCTTTTTACGAAGCAGTGACAAAAACCTATCACGGCAGACCTTATGATGCACAGATAGAGATTCTTAAAGAGCTTTGCGAACGGTTTCCTATAGACTCT
enoA_18   ATGACTGCAATAGAAGCCGCAGGTTTTGTCCCTGGGGAGGAAGTTTCTTTAGCTCTAGATTGCGCAGCATCTTCTTTTTACGAAGCAGCGACAAAAACCTATCACGGCAGACCTTATGATGCACAGATAGAGATTCTTAAAGAGCTTTGCGAGCGGTTTCCTATAGACTCT
enoA_22   ATGACTGCAATAGAAGCCGCAGGTTTTGTCCCTGGGGAGGAAGTTTCTTTAGCTCTAGATTGCGCAGCATCTTCTTTTTACGAAGCAGCGACAAAAACCTATCACGGCAGACCTTATGATGCACAAATAGAGATTCTTAAAGAGCTTTGCGAGCGGTTTCCTATAGACTCT
enoA_23   ATGACTGCAATAGAAGCCGCAGGTTTTGTCCCTGGGGAGGAAGTTTCTTTAGCTCTAGATTGCGCAGCATCTTCTTTTTACGAAGCAGCGACAAAAACCTATCACGGCAGACCTTATGATGCACAGATAGAGATTCTTAAAGAGCTTTGCGAGCGGTTTCCTATAGACTCT
enoA_19   ATGACTGCAATAGAAGCCGCAGGTTTTGTCCCTGGGGAGGAAGTTTCTTTAGCTCTAGATTGCGCAGCATCTTCTTTTTACGAAGCAGCGACAAAAACCTATCACGGTAGACCTTATGATGCACAGATAGAGATTCTTAAAGAGCTTTGCGAGCGGTTTCCTATAGACTCT
enoA_20   ATGACTGCAATAGAAGCCGCAGGTTTTGTCCCTGGGGAGGAGGTTTCTTTAGCTCTAGATTGCGCAGCATCTTCTTTTTACGAAGCAGCGACAAAAACCTATCACGGTAGACCTTATGATGCACAGATAGAGATTCTTAAAGAGCTTTGCGAGCGGTTTCCTATAGACTCT
enoA_21   ATGACTGCAATAGAAGCCGCAGGTTTTGTCCCTGGGGAGGAGGTTTCTTTAGCTCTAGATTGCGCAGCATCTTCTTTTTACGAAGCAGCGACAAAAACCTATCACGGTAGACCTTATGATGCACAGATAGAGATTCTTAAAGAGCTTTGCGAGCGGTTTCCTATAGACTCT
enoA_11   TTGGCTGCTATTGAAAAGGCTGGCTTTATCCCTGGAGAGGATATTTCTTTAGCTCTTGATTGCGCAGCTTCCTCGTTTTACAATACTCAGGATAAAACTTATGATGGCAAGTCCGCTGCAGATCAAGTTGCTGTACTCACAGAATTATGCGATCGCTATCCTATAGATTCT
enoA_2    TTAACAGCAATCGAAACTGCAGGATTCACACCTAGAGAAGATATTTCTTTAGCTCTCGACTGCGCTGCTTCTTCTTTCTATAATACCCAAGATAAAACCTATGATGGGAAATCGTATGCAGATCAAGTGGGTATACTTGCAGAACTCTGTGAGCACTATCCTATAGATTCT
enoA_3    TTAACAGCAATCGAAACTGCAGGATTCACACCTAGAGAAGATATTTCTTTAGCTCTCGACTGCGCTGCTTCTTCTTTCTATAATACCCAAGATAAAACCTATGATGGGAAATCGTATGCAGATCAAGTGGGTATACTTGCAGAACTCTGTGAGCACTATCCTATAGATTCT
enoA_25   TTAACAGCAATCGAAACTGCAGGATTCACACCCAGAGAAGATATTTCTTTAGCTCTCGACTGCGCTGCTTCTTCTTTCTATAATACCCAAGATAAAACCTATGATGGGAAATCGTATGCAGATCAAGTGGGTATACTTGCAGAACTCTGTGAGCACTATCCTATAGATTCT
enoA_4    TTAACAGCAATCGAAACTGCAGGATTCACACCTAGAGAAGATATTTCTTTAGCTCTCGACTGCGCTGCTTCTTCTTTCTATAATACCCAAGATAAAACCTATGATGGGAAATCGTATGCAGATCAAGTGGGTATACTTGCAGAACTCTGTGAGCACTATCCTATAGATTCT
enoA_1    TTAACAGCAATCGAAACTGCAGGATTCACACCTAGAGAAGATATTTCTTTAGCTCTCGACTGCGCTGCTTCTTCTTTCTATAATACCCAAGATAAAACCTATGATGGGAAATCGTATGCAGATCAAGTGGGTATACTTGCAGAACTCTGTGAGCACTATCCTATAGATTCT
enoA_24   TTAACAGCAATCGAAACTGCAGGATTCACACCTAGAGAAGATATTTCTTTAGCTCTCGACTGCGCTGCTTCTTCTTTCTATAATACCCAAGATAAAACCTATGATGGGAAATCGTATGCAGATCAAGTGGGTATACTTGCAGAACTCTGTGAGCACTATCCTATAGATTCT
```

D. Amino-acid substitutions among *enoA*-encoded protein alleles (b)

```
                                                                                                             111111111111111111111111111
                  1111111111222222222233333333334444444444555555555566666666667777777777888888888899999999990000000000111111111122222222
         1234567890123456789012345678901234567890123456789012345678901234567890123456789012345678901234567890123456789012345678901234567
enoA_8   GMHANNGLQFQEFMIRPIGAHSLQEAVRMGADVFHTLKNLLNDKHLATGVGDEGGFAPQLKSNSEALDLLVLAIEKAGFQPGEEISLALDCAASSFYDTKTETYAGKNSQEQVGILADLCDRYPIDS
enoA_16  GMHANNGLQFQEFMIRPIGAHSLQEAVRMGADVFHTLKNLLNDKHLATGVGDEGGFAPQLKSNSEALDLLVLAIEKAGFQPGEEISLALDCAASSFYDTKTETYAEKNSQEQVGILADLCDRYPIDS
enoA_14  GMHANNGLQFQEFMIRPIGAHSLQEAVRMGADVFHTLKNLLNDKHLATGVGDEGGFAPQLKSNSEALDLLVLAIEKAGFQPGEEISLALDCAASSFYDTKTETYAGKNSQEQVGILADLCDRYPIDS
enoA_13  GMHADNGLQFQEFMIRPIGAHSLKEAVRMGADVFHALKKLLNDRHLATGVGDEGGFAPNLTSNSEALDLLLLAIEKAGFQPGEEISLALDCAASSFYDTKTKTYEGKNSQEQVSILADLCDRYPIDS
enoA_15  GMHADNGLQFQEFMIRPIGAHSLKEAVRMGADVFHALKKLLNDRHLATGVGDEGGFAPNLTSNSEALDLLLLAIEKAGFQPGEEISLALDCAASSFYDTKTKTYEGKNSQEQVSILADLCDRYPIDS
enoA_10  GMHANNGLQFQEFMIRPIGATSIKEAVRMGADVFHTLKKLLNDKNLATGVGDEGGFAPQLKSNSEALDLLVLAIEKSGFQPGKEISLALDCAASSFYDTKTKTYEGKSYQEQVEILADLCDRYPIDS
enoA_9   GMHANNGLQFQEFMIRPIGATSLKEAVRMGADVFHSLKNILNEKNLATGVGDEGGFAPQLKSNSEALDLLVLAIEKAGFQPGEDISLALDCAASSFYDTKAETYDGKSYEEQVNILADLCDRYPIDS
enoA_5   GMHADNGLGFQEFMIRPIGASSIKEAVNMGADVFHTLKKLLHERGLSTGVGDEGGFAPNLASNEEALELLLLAIEKAGFTPGKDISLALDCAASSFYNVKTGTYDGRHYEEQIAILSNLCDRYPIDS
enoA_6   GMHADNGLEFQEFMIRPIGASSIKEAVNMGADVFHTLKKLLHERGLSTGVGDEGGFAPNLASNEEALELLLLAIEKAGFTPGKDISLALDCAASSFYNVKTGTYDGRHYEEQIAILSNLCDRYPIDS
enoA_7   GMHADNGLEFQEFMIRPIGASSIKEAVNMGADVFHTLKKLLHERGLSTGVGDEGGFAPNLASNEEALELLLLAIEKAGFTPGKDISLALDCAASSFYNVKTGTYDGRHYEEQIAILSNLCDRYPIDS
enoA_17  GMHADNGLEFQEFMIRPIGASSIKEAVNMGADVFHTLKKLLHERGLSTGVGDEGGFAPNLASNEEALELLLLAIEKAGFTPGKDISLALDCAASSFYNVKTGTYDGRHYEEQIAILSNLCDRYPIDS
enoA_12  GMHADNGLEFQEFMIRPIGAHSMHEAVSMGADVFHALKNLLKSRHLATGVGDEGGFAPNLRSNAEALELLMTAIEAAGFVPGEEVSLALDCAASSFYEAVTKTYHGRPYDAQIEILKELCERFPIDS
enoA_18  GMHADNGLEFQEFMIRPIGAHSMHEAVSMGADVFHALKNLLKSRHLATGVGDEGGFAPNLRSNAEALELLMTAIEAAGFVPGEEVSLALDCAASSFYEAATKTYHGRPYDAQIEILKELCERFPIDS
enoA_22  GMHADNGLEFQEFMIRPIGAHSMHEAVSMGADVFHALKNLLKSRHLATGVGDEGGFAPNLRSNAEALELLMTAIEAAGFVPGEEVSLALDCAASSFYEAATKTYHGRPYDAQIEILKELCERFPIDS
enoA_23  GMHADNGLEFQEFMIRPIGAHSMHEAVSMGADVFHALKNLLKSRHLATGVGDEGGFAPNLRSNAEALELLMTAIEAAGFVPGEEVSLALDCAASSFYEAATKTYHGRPYDAQIEILKELCERFPIDS
enoA_19  GMHADNGLEFQEFMIRPIGAHSMHEAVSMGADVFHALKNLLKSRHLATGVGDEGGFAPNLRSNAEALELLMTAIEAAGFVPGEEVSLALDCAASSFYEAATKTYHGRPYDAQIEILKELCERFPIDS
enoA_20  GMHADNGLEFQEFMIRPIGAHSMHEAVSMGADVFHALKNLLKSRHLATGVGDEGGFAPNLRSNAEALELLMTAIEAAGFVPGEEVSLALDCAASSFYEAATKTYHGRPYDAQIEILKELCERFPIDS
enoA_21  GMHADNGLEFQEFMIRPIGAHSMHEAVSMGADVFHALKNLLKSRHLATGVGDEGGFAPNVRSNAEALELLMTAIEAAGFVPGEEVSLALDCAASSFYEAATKTYHGRPYDAQIEILKELCERFPIDS
enoA_11  GMHATNGLQFQEFMIRPISAPSLTEAVRMGAEVFHTLKKILQNRQLSTGVGDEGGFAPQLASNSEALDLLLAAIEKAGFIPGEDISLALDCAASSFYNTQDKTYDGKSAADQVAVLTELCDRYPIDS
enoA_2   GMHATNGLQFQEFMIRPISAPSLKEAVRMGAEVFNALKKILQNRQLATGVGDEGGFAPNLASNAEALDLLLTAIETAGFTPREDISLALDCAASSFYNTQDKTYDGKSYADQVGILAELCEHYPIDS
enoA_3   GMHATNGLQFQEFMIRPISAPSLTEAVRMGAEVFNALKKILQNRQLATGVGDEGGFAPNLASNAEALDLLLTAIETAGFTPREDISLALDCAASSFYNTQDKTYDGKSYADQVGILAELCEHYPIDS
enoA_25  GMHATNGLQFQEFMIRPISAPSLTEAVRMGAEVFNALKKILQNRQLATGVGDEGGFAPNLASNAEALDLLLTAIETAGFTPREDISLALDCAASSFYNTQDKTYDGKSYADQVGILAELCEHYPIDS
enoA_4   GMHATNGLQFQEFMIRPISAPSLTEAVRMGAEVFNALKKILQNRQLATGVGDEGGFAPNLASNAEALDLLLTAIETAGFTPREDISLALDCAASSFYNTQDKTYDGKSYADQVGILAELCEHYPIDS
enoA_1   GMHATNGLQFQEFMIRPISAPSLTEAVRMGAEVFNALKKILQNRQLATGVGDEGGFAPNLASNAEALDLLLTAIETAGFTPREDISLALDCAASSFYNTQDKTYDGKSYADQVGILAELCEHYPIDS
enoA_24  GMHATNGLQFQEFMIRPISAPSLTEAVRMGAEVFNALKKILQNRQLATGVGDEGGFAPNLASNAEALDLLLTAIETAGFTPREDISLALDCAASSFYNTQDKTYDGKSYADQVGILAELCEHYPIDS
```
