# Supplementary material for: Diversification and Distribution of Ruminant Chlamydia abortus Clones Assessed by MLST and MLVA
Source: PLoS One. 2015 May 22;10(5):e0126433. doi: 10.1371/journal.pone.0126433 (PMC4441495; doi:10.1371/journal.pone.0126433)
Supplement: S1 Table — (PDF) [file pone.0126433.s006.pdf]

S1 Table. Variant alleles of the single-locus variants (SLVs) found within *C. abortus* clonal complex and comparison with LLG singleton clone (ST30).

**ST of ancestor clone**	ST of SLV	Variant locus in SLV	Ancestral allele	SLV allele	No of nucleotide differences	Other clones containing SLV allele	Amino acid change[a]
19	86	*hemN*	4	14	1	ST25	Syn
19	87	*hemN*	4	21	1	None	A-T [b]
19	29	*fumC*	5	18	1	None	Syn
86	25	*gatA*	5	18	1	None	Syn
**comparison with ST30 (singleton clone)**		Variant locus in singleton	ST19 allele	Singleton allele		Other clones containing singleton allele	
		gatA	5	13	1	ST36*	G-D [c]
		oppA	8	18	1	None	Syn
		gidA	8	19	1	None	Syn
		enoA	8	16	2	None	G-E [d]
		fumC	5	17	3[e]	None	Syn

[a] Residue in ST19 is given first. Syn, synonymous change

[b] Amino-acid Thr (T) at the position 115 is unique among all chlamydial *hemN*-encoded protein alleles (S2A.b Fig)

[c] Amino-acid Gly (G) characterizing *C. abortus* clonal complex is unique among all chlamydial *gatA*-encoded protein alleles at the conserved position 125 (S2B.b Fig)

[d] Amino-acid Glu (E) is unique among all chlamydial *enoA*-encoded protein alleles at the conserved position 106 (S2D.b Fig)

[e] Residue T at position 243 of *fumC*_17 harbouring in the LLG singleton is unique among all chlamydial *fumC* alleles (S2C.b Fig)

* ST36 corresponds to the variant *C. psittaci* 84/2334 strain previously proposed belonging to *C. abortus* [22]
