# Supplementary material for: Diversification and Distribution of Ruminant Chlamydia abortus Clones Assessed by MLST and MLVA
Source: PLoS One. 2015 May 22;10(5):e0126433. doi: 10.1371/journal.pone.0126433 (PMC4441495; doi:10.1371/journal.pone.0126433)
Supplement: S2 Table — (PDF) [file pone.0126433.s007.pdf]

S2 Table. Codon-based test of neutrality (a) and positive (b) or purifying (c) selection among *hemN*-locus sequences.

a.

	hemN_1	hemN_2	hemN_3	**hemN_4**	hemN_5	hemN_6	hemN_7	hemN_8	hemN_9	hemN_10	hemN_11	hemN_12	hemN_13	**hemN_14**	hemN_15	hemN_16	hemN_17	hemN_18	hemN_19	**hemN_21**
hemN_1		[0.98]	[-8.23]	[-9.11]	[-9.24]	[-9.23]	[-10.00]	[-5.72]	[-9.24]	[-9.24]	[-9.21]	[-9.17]	[-9.14]	[-8.97]	[1.40]	[-7.75]	[-5.72]	[1.42]	[-0.68]	[-9.04]
hemN_2	0.33		[-8.28]	[-9.33]	[-9.34]	[-9.30]	[-10.20]	[-5.72]	[-9.33]	[-9.33]	[-9.30]	[-9.26]	[-9.23]	[-9.19]	[0.98]	[-7.79]	[-5.72]	[1.00]	[-1.05]	[-9.25]
hemN_3	0.00	0.00		[-8.99]	[-10.27]	[-9.21]	[-10.04]	[-9.82]	[-9.32]	[-9.23]	[-9.30]	[-9.22]	[-9.30]	[-9.71]	[-8.32]	[-1.53]	[-8.64]	[-8.26]	[-7.96]	[-8.97]
**hemN_4**	0.00	0.00	0.00		[-9.31]	[-8.85]	[-9.29]	[-10.23]	[-5.35]	[-5.35]	[-5.23]	[-5.24]	[-5.24]	[-1.07]	[-9.37]	[-8.52]	[-9.98]	[-9.13]	[-9.13]	[1.02]
hemN_5	0.00	0.00	0.00	0.00		[-9.95]	[-10.57]	[-11.22]	[-9.53]	[-9.53]	[-9.49]	[-9.48]	[-9.48]	[-9.34]	[-9.38]	[-9.97]	[-11.01]	[-9.30]	[-9.48]	[-9.31]
hemN_6	0.00	0.00	0.00	0.00	0.00		[-9.41]	[-11.00]	[-8.17]	[-8.00]	[-8.13]	[-8.01]	[-8.18]	[-8.71]	[-9.34]	[-9.55]	[-10.69]	[-9.28]	[-9.15]	[-8.85]
hemN_7	0.00	0.00	0.00	0.00	0.00	0.00		[-9.94]	[-8.22]	[-8.22]	[-8.16]	[-8.24]	[-8.14]	[-9.26]	[-10.15]	[-9.79]	[-9.73]	[-10.02]	[-10.44]	[-9.22]
hemN_8	0.00	0.00	0.00	0.00	0.00	0.00	0.00		[-9.39]	[-9.63]	[-9.37]	[-9.65]	[-9.36]	[-10.30]	[-5.73]	[-9.82]	[-1.06]	[-5.72]	[-6.79]	[-10.24]
hemN_9	0.00	0.00	0.00	0.00	0.00	0.00	0.00	0.00		[-1.10]	[-0.99]	[-0.75]	[0.99]	[-5.20]	[-9.38]	[-9.36]	[-9.12]	[-9.15]	[-9.16]	[-5.34]
hemN_10	0.00	0.00	0.00	0.00	0.00	0.00	0.00	0.00	0.27		[-1.46]	[1.01]	[-0.75]	[-5.20]	[-9.38]	[-9.27]	[-9.36]	[-9.15]	[-9.16]	[-5.34]
hemN_11	0.00	0.00	0.00	0.00	0.00	0.00	0.00	0.00	0.32	0.15		[-1.21]	[-0.68]	[-5.07]	[-9.35]	[-9.34]	[-9.09]	[-9.12]	[-9.13]	[-5.22]
hemN_12	0.00	0.00	0.00	0.00	0.00	0.00	0.00	0.00	0.46	0.32	0.23		[-0.43]	[-5.08]	[-9.31]	[-9.27]	[-9.37]	[-9.09]	[-9.09]	[-5.23]
hemN_13	0.00	0.00	0.00	0.00	0.00	0.00	0.00	0.00	0.32	0.45	0.50	0.67		[-5.10]	[-9.29]	[-9.35]	[-9.09]	[-9.06]	[-9.07]	[-5.23]
**hemN_14**	0.00	0.00	0.00	**0.29**	0.00	0.00	0.00	0.00	0.00	0.00	0.00	0.00	0.00		[-9.24]	[-9.13]	[-10.04]	[-9.00]	[-9.01]	[-0.74]
hemN_15	0.16	0.33	0.00	0.00	0.00	0.00	0.00	0.00	0.00	0.00	0.00	0.00	0.00	0.00		[-7.83]	[-5.73]	[1.44]	[-0.69]	[-9.30]
hemN_16	0.00	0.00	0.13	0.00	0.00	0.00	0.00	0.00	0.00	0.00	0.00	0.00	0.00	0.00	0.00		[-9.25]	[-7.77]	[-7.88]	[-8.52]
hemN_17	0.00	0.00	0.00	0.00	0.00	0.00	0.00	0.29	0.00	0.00	0.00	0.00	0.00	0.00	0.00	0.00		[-5.72]	[-6.79]	[-9.98]
hemN_18	0.16	0.32	0.00	0.00	0.00	0.00	0.00	0.00	0.00	0.00	0.00	0.00	0.00	0.00	0.15	0.00	0.00		[-0.68]	[-9.05]
hemN_19	0.50	0.30	0.00	0.00	0.00	0.00	0.00	0.00	0.00	0.00	0.00	0.00	0.00	0.00	0.49	0.00	0.00	0.50		[-9.05]
**hemN_21**	0.00	0.00	0.00	**0.31**	0.00	0.00	0.00	0.00	0.00	0.00	0.00	0.00	0.00	**0.46**	0.00	0.00	0.00	0.00	0.00	

Analysis:Z-test of Selection;Scope:in Sequence Pairs; **Test Hypothesis (HA: alternative): Neutrality (HA: dN =/= dS)**; Variance Estimation Method: Bootstrap method; No. of Bootstrap Replications: 500; Substitutions Type: Syn-Nonsynonymous; Model/Method: Nei-Gojobori method (Proportion); Gaps/Missing Data Treatment: Pairwise deletion; No. of Sites: 144; Prob: Probability computed (must be <0.05 for hypothesis rejection at 5% level [yellow backgroud]); Stat (right): Statistic used to compute the probability.

The probability of rejecting the null hypothesis of strict-neutrality (dN = dS) (below diagonal) is shown. Values of P less than 0.05 are considered significant at the 5% level and are highlighted. The test statistic (dN - dS) is shown above the diagonal. dS and dN are the numbers of synonymous and nonsynonymous substitutions per site, respectively. The variance of the difference was computed using the bootstrap method (500 replicates). Analyses were conducted using the Nei-Gojobori method [31]. The analysis involved 20 nucleotide sequences. All ambiguous positions were removed for each sequence pair. There were a total of 144 positions in the final dataset. Evolutionary analyses were conducted in MEGA5 [30].

b.

	hemN_1	hemN_2	hemN_3	**hemN_4**	hemN_5	hemN_6	hemN_7	hemN_8	hemN_9	hemN_10	hemN_11	hemN_12	hemN_13	**hemN_14**	hemN_15	hemN_16	hemN_17	hemN_18	hemN_19	**hemN_21**
hemN_1		[1.00]	[-9.09]	[-9.49]	[-9.61]	[-10.33]	[-11.01]	[-5.80]	[-9.80]	[-9.80]	[-9.77]	[-9.76]	[-9.69]	[-9.36]	[1.42]	[-9.17]	[-5.80]	[1.45]	[-0.66]	[-9.42]
hemN_2	0.16		[-9.15]	[-9.56]	[-9.61]	[-10.39]	[-11.07]	[-5.81]	[-9.86]	[-9.86]	[-9.83]	[-9.82]	[-9.75]	[-9.43]	[1.04]	[-9.23]	[-5.81]	[1.02]	[-1.00]	[-9.48]
hemN_3	1.00	1.00		[-8.86]	[-10.67]	[-9.77]	[-11.35]	[-9.74]	[-10.28]	[-10.04]	[-10.24]	[-10.11]	[-10.23]	[-9.51]	[-9.20]	[-1.58]	[-9.25]	[-9.12]	[-9.33]	[-8.85]
**hemN_4**	1.00	1.00	1.00		[-9.33]	[-9.58]	[-9.44]	[-10.62]	[-5.41]	[-5.41]	[-5.27]	[-5.31]	[-5.29]	[-1.03]	[-9.60]	[-7.93]	[-10.50]	[-9.44]	[-9.34]	[1.04]
hemN_5	1.00	1.00	1.00	1.00		[-9.21]	[-11.27]	[-11.53]	[-9.43]	[-9.43]	[-9.40]	[-9.37]	[-9.34]	[-9.24]	[-9.64]	[-10.36]	[-11.40]	[-9.57]	[-9.81]	[-9.33]
hemN_6	1.00	1.00	1.00	1.00	1.00		[-10.44]	[-10.51]	[-9.00]	[-8.81]	[-8.95]	[-8.83]	[-8.99]	[-9.46]	[-10.45]	[-10.48]	[-10.32]	[-10.35]	[-10.14]	[-9.58]
hemN_7	1.00	1.00	1.00	1.00	1.00	1.00		[-10.48]	[-8.30]	[-8.30]	[-8.31]	[-8.31]	[-8.21]	[-9.38]	[-11.02]	[-11.16]	[-10.30]	[-10.87]	[-11.30]	[-9.37]
hemN_8	1.00	1.00	1.00	1.00	1.00	1.00	1.00		[-9.72]	[-9.96]	[-9.69]	[-9.96]	[-9.71]	[-10.64]	[-5.81]	[-9.72]	[-1.06]	[-5.64]	[-7.26]	[-10.58]
hemN_9	1.00	1.00	1.00	1.00	1.00	1.00	1.00	1.00		[-0.99]	[-1.01]	[-0.66]	[1.04]	[-5.29]	[-9.90]	[-10.49]	[-9.61]	[-9.73]	[-9.58]	[-5.43]
hemN_10	1.00	1.00	1.00	1.00	1.00	1.00	1.00	1.00	1.00		[-1.50]	[1.01]	[-0.68]	[-5.29]	[-9.90]	[-10.24]	[-9.86]	[-9.73]	[-9.58]	[-5.43]
hemN_11	1.00	1.00	1.00	1.00	1.00	1.00	1.00	1.00	1.00	1.00		[-1.23]	[-0.68]	[-5.13]	[-9.87]	[-10.46]	[-9.58]	[-9.69]	[-9.54]	[-5.28]
hemN_12	1.00	1.00	1.00	1.00	1.00	1.00	1.00	1.00	1.00	0.16	1.00		[-0.38]	[-5.17]	[-9.86]	[-10.31]	[-9.86]	[-9.69]	[-9.54]	[-5.33]
hemN_13	1.00	1.00	1.00	1.00	1.00	1.00	1.00	1.00	0.15	1.00	1.00	1.00		[-5.16]	[-9.79]	[-10.44]	[-9.60]	[-9.62]	[-9.47]	[-5.31]
**hemN_14**	1.00	1.00	1.00	**1.00**	1.00	1.00	1.00	1.00	1.00	1.00	1.00	1.00	1.00		[-9.46]	[-7.91]	[-10.51]	[-9.31]	[-9.19]	[-0.69]
hemN_15	0.08	0.15	1.00	1.00	1.00	1.00	1.00	1.00	1.00	1.00	1.00	1.00	1.00	1.00		[-9.28]	[-5.81]	[1.46]	[-0.66]	[-9.52]
hemN_16	1.00	1.00	1.00	1.00	1.00	1.00	1.00	1.00	1.00	1.00	1.00	1.00	1.00	1.00	1.00		[-9.99]	[-9.20]	[-9.42]	[-7.92]
hemN_17	1.00	1.00	1.00	1.00	1.00	1.00	1.00	1.00	1.00	1.00	1.00	1.00	1.00	1.00	1.00	1.00		[-5.64]	[-7.26]	[-10.46]
hemN_18	0.07	0.16	1.00	1.00	1.00	1.00	1.00	1.00	1.00	1.00	1.00	1.00	1.00	1.00	0.07	1.00	1.00		[-0.67]	[-9.36]
hemN_19	1.00	1.00	1.00	1.00	1.00	1.00	1.00	1.00	1.00	1.00	1.00	1.00	1.00	1.00	1.00	1.00	1.00	1.00		[-9.26]
**hemN_21**	1.00	1.00	1.00	**0.15**	1.00	1.00	1.00	1.00	1.00	1.00	1.00	1.00	1.00	**1.00**	1.00	1.00	1.00	1.00	1.00	

Analysis:Z-test of Selection;Scope:in Sequence Pairs; **Test Hypothesis (HA: alternative): Positive selection (HA: dN > dS)**; Variance Estimation Method: Bootstrap method; No. of Bootstrap Replications: 500; Substitutions Type: Syn-Nonsynonymous; Model/Method: Nei-Gojobori method (Proportion); Gaps/Missing Data Treatment: Pairwise deletion; No. of Sites: 144; Prob: Probability computed (must be <0.05 for hypothesis rejection at 5% level [yellow backgroud]); Stat (right): Statistic used to compute the probability.

The probability of rejecting the null hypothesis of strict-neutrality (dN = dS) in favor of the alternative hypothesis (dN > dS) (below diagonal) is shown. Values of P less than 0.05 are considered significant at the 5% level and are highlighted. The test statistic (dN - dS) is shown above the diagonal. dS and dN are the numbers of synonymous and nonsynonymous substitutions per site, respectively.

c.

	hemN_1	hemN_2	hemN_3	**hemN_4**	hemN_5	hemN_6	hemN_7	hemN_8	hemN_9	hemN_10	hemN_11	hemN_12	hemN_13	**hemN_14**	hemN_15	hemN_16	hemN_17	hemN_18	hemN_19	**hemN_21**
hemN_1		[-0.99]	[9.31]	[8.94]	[9.45]	[9.69]	[10.32]	[7.29]	[9.11]	[9.11]	[9.08]	[9.04]	[9.02]	[8.91]	[-1.56]	[9.45]	[7.29]	[-1.45]	[0.65]	[8.87]
hemN_2	1.00		[9.36]	[9.05]	[9.49]	[9.76]	[10.45]	[7.32]	[9.18]	[9.18]	[9.15]	[9.11]	[9.09]	[9.02]	[-1.07]	[9.50]	[7.32]	[-1.02]	[0.98]	[8.98]
hemN_3	0.00	0.00		[10.64]	[10.32]	[9.71]	[10.72]	[11.01]	[10.62]	[10.53]	[10.59]	[10.52]	[10.60]	[10.67]	[9.43]	[1.56]	[11.38]	[9.33]	[9.58]	[10.62]
**hemN_4**	0.00	0.00	0.00		[9.17]	[9.35]	[9.02]	[10.90]	[5.27]	[5.27]	[5.23]	[5.16]	[5.16]	[1.04]	[9.13]	[11.10]	[10.73]	[8.89]	[8.91]	[-1.02]
hemN_5	0.00	0.00	0.00	0.00		[9.21]	[10.45]	[11.71]	[9.33]	[9.33]	[9.29]	[9.26]	[9.22]	[9.01]	[9.60]	[10.01]	[11.48]	[9.45]	[9.64]	[9.17]
hemN_6	0.00	0.00	0.00	0.00	0.00		[9.86]	[10.31]	[8.71]	[8.57]	[8.67]	[8.57]	[8.70]	[9.17]	[9.83]	[10.10]	[10.05]	[9.73]	[9.60]	[9.35]
hemN_7	0.00	0.00	0.00	0.00	0.00	0.00		[10.52]	[8.02]	[8.02]	[7.98]	[8.02]	[7.95]	[8.93]	[10.38]	[10.48]	[10.29]	[10.29]	[10.75]	[8.95]
hemN_8	0.00	0.00	0.00	0.00	0.00	0.00	0.00		[9.33]	[9.52]	[9.29]	[9.52]	[9.27]	[10.82]	[7.34]	[11.00]	[1.03]	[7.31]	[9.40]	[10.89]
hemN_9	0.00	0.00	0.00	0.00	0.00	0.00	0.00	0.00		[1.00]	[1.01]	[0.68]	[-1.03]	[5.03]	[9.24]	[10.82]	[9.13]	[9.02]	[8.99]	[5.25]
hemN_10	0.00	0.00	0.00	0.00	0.00	0.00	0.00	0.00	0.16		[1.40]	[-0.99]	[0.69]	[5.03]	[9.24]	[10.74]	[9.33]	[9.02]	[8.99]	[5.25]
hemN_11	0.00	0.00	0.00	0.00	0.00	0.00	0.00	0.00	0.16	0.08		[1.18]	[0.69]	[4.98]	[9.21]	[10.78]	[9.09]	[8.98]	[8.96]	[5.20]
hemN_12	0.00	0.00	0.00	0.00	0.00	0.00	0.00	0.00	0.25	1.00	0.12		[0.39]	[4.92]	[9.17]	[10.73]	[9.33]	[8.95]	[8.92]	[5.13]
hemN_13	0.00	0.00	0.00	0.00	0.00	0.00	0.00	0.00	1.00	0.25	0.25	0.35		[4.93]	[9.15]	[10.79]	[9.06]	[8.93]	[8.90]	[5.14]
**hemN_14**	0.00	0.00	0.00	**0.15**	0.00	0.00	0.00	0.00	0.00	0.00	0.00	0.00	0.00		[9.10]	[11.11]	[10.64]	[8.87]	[8.87]	[0.70]
hemN_15	1.00	1.00	0.00	0.00	0.00	0.00	0.00	0.00	0.00	0.00	0.00	0.00	0.00	0.00		[9.57]	[7.34]	[-1.50]	[0.65]	[9.06]
hemN_16	0.00	0.00	0.06	0.00	0.00	0.00	0.00	0.00	0.00	0.00	0.00	0.00	0.00	0.00	0.00		[11.31]	[9.46]	[9.70]	[11.08]
hemN_17	0.00	0.00	0.00	0.00	0.00	0.00	0.00	0.15	0.00	0.00	0.00	0.00	0.00	0.00	0.00	0.00		[7.31]	[9.40]	[10.72]
hemN_18	1.00	1.00	0.00	0.00	0.00	0.00	0.00	0.00	0.00	0.00	0.00	0.00	0.00	0.00	1.00	0.00	0.00		[0.65]	[8.82]
hemN_19	0.26	0.16	0.00	0.00	0.00	0.00	0.00	0.00	0.00	0.00	0.00	0.00	0.00	0.00	0.26	0.00	0.00	0.26		[8.85]
**hemN_21**	0.00	0.00	0.00	**1.00**	0.00	0.00	0.00	0.00	0.00	0.00	0.00	0.00	0.00	**0.24**	0.00	0.00	0.00	0.00	0.00	

Analysis:Z-test of Selection;Scope:in Sequence Pairs; **Test Hypothesis (HA: alternative): Purifying selection (HA: dN < dS)**; Variance Estimation Method: Bootstrap method; No. of Bootstrap Replications: 500; Substitutions Type: Syn-Nonsynonymous; Model/Method: Nei-Gojobori method (Proportion); Gaps/Missing Data Treatment: Pairwise deletion; No. of Sites: 144; Prob: Probability computed (must be <0.05 for hypothesis rejection at 5% level [yellow backgroud]); Stat (right): Statistic used to compute the probability.

The probability of rejecting the null hypothesis of strict-neutrality (dN = dS) in favor of the alternative hypothesis (dN < dS) (below diagonal) is shown. Values of P less than 0.05 are considered significant at the 5% level and are highlighted. The test statistic (dS - dN) is shown above the diagonal. dS and dN are the numbers of synonymous and nonsynonymous substitutions per site, respectively.
